# Supplementary figures and images for: NIa-Pro of sugarcane mosaic virus targets Corn Cysteine Protease 1 (CCP1) to undermine salicylic acid-mediated defense in maize
Source: PLoS Pathog. 2024 Mar 14;20(3):e1012086. doi: 10.1371/journal.ppat.1012086 (PMC10965072; doi:10.1371/journal.ppat.1012086)

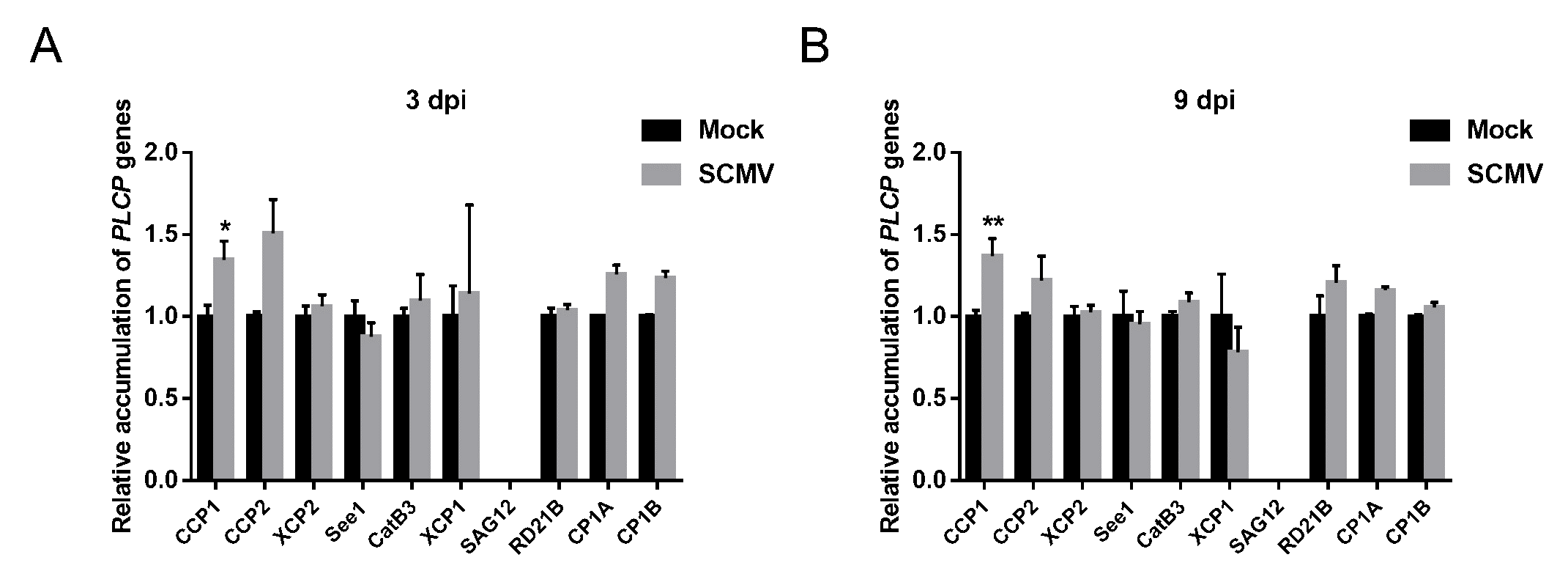

Supplement: S1 Fig — A) Relative expression of maize PLCPs at 3 days post inoculation (dpi). B) Relative expression of maize PLCPs at 9 dpi. (TIF) [file ppat.1012086.s002.tif]

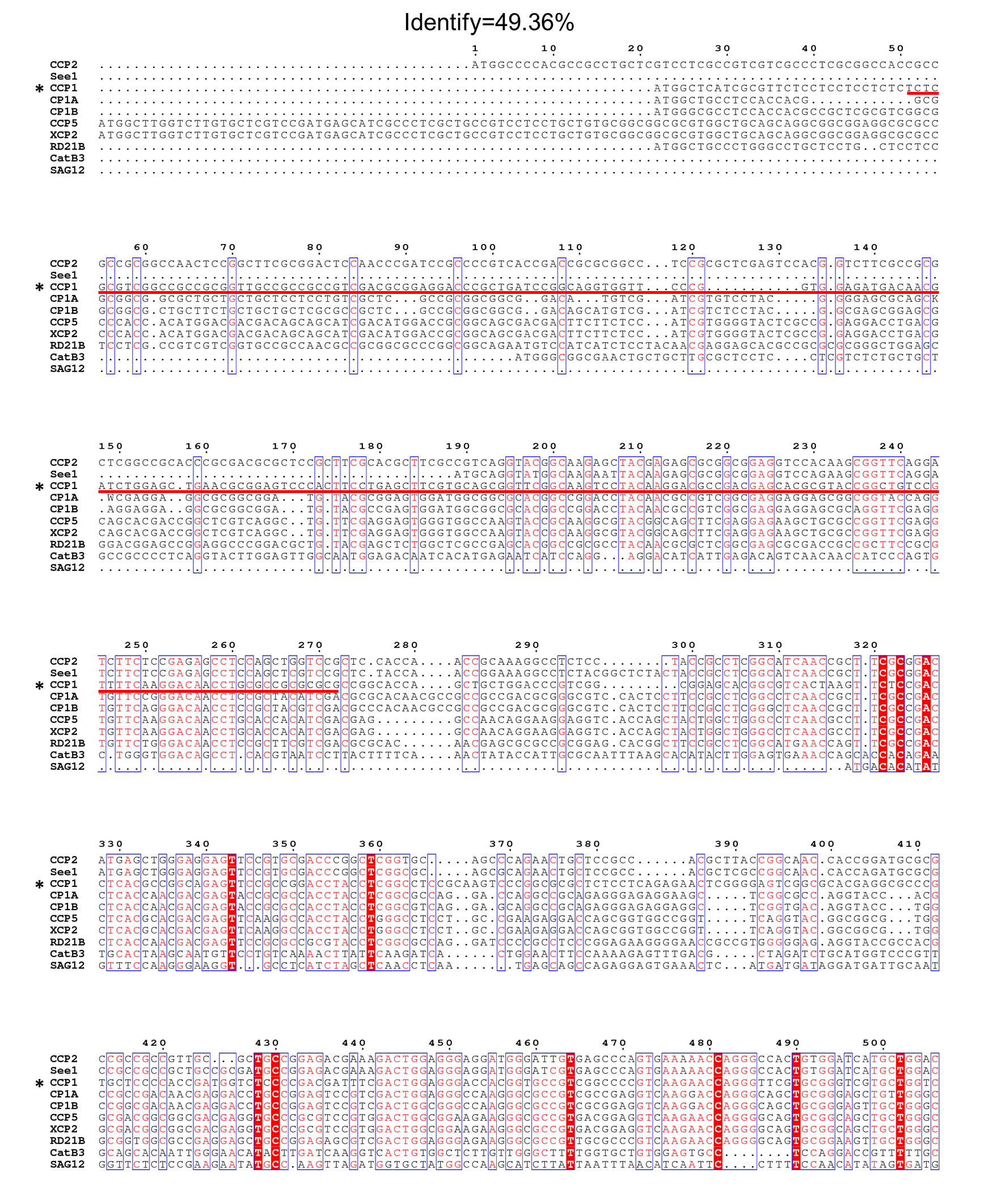

Supplement: S2 Fig — The redlined nucleotide sequence is the partial CCP1 sequence inserted into the CMV-based VIGS vectors for specific silencing of CCP1. (TIF) [file ppat.1012086.s003.tif]

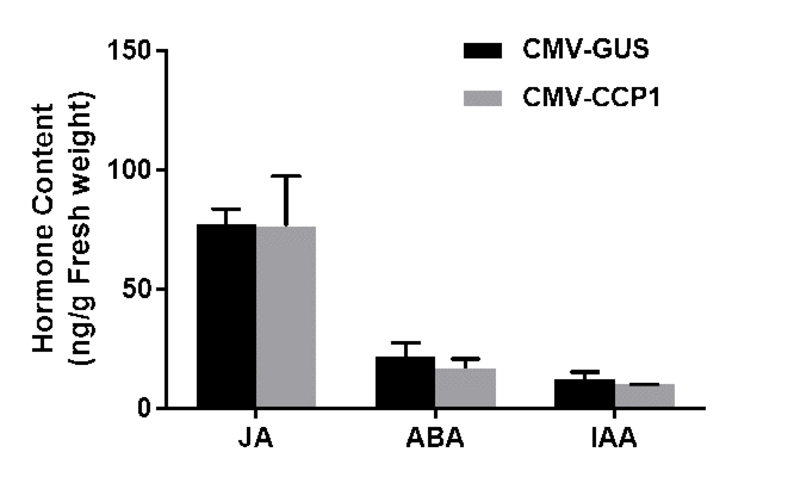

Supplement: S3 Fig — (TIF) [file ppat.1012086.s004.tif]

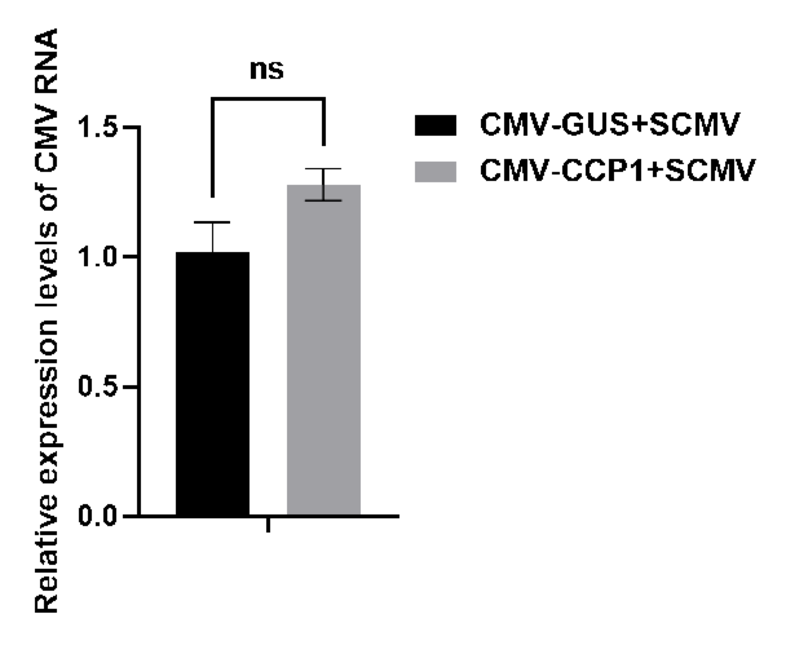

Supplement: S4 Fig — The coat protein (cp) gene of CMV RNA3 was used for RT-qPCR analysis. The statistical results evaluated by Student’s t test analysis indicate that the differences between the two are not significant (ns, P > 0.05). (TIF) [file ppat.1012086.s005.tif]

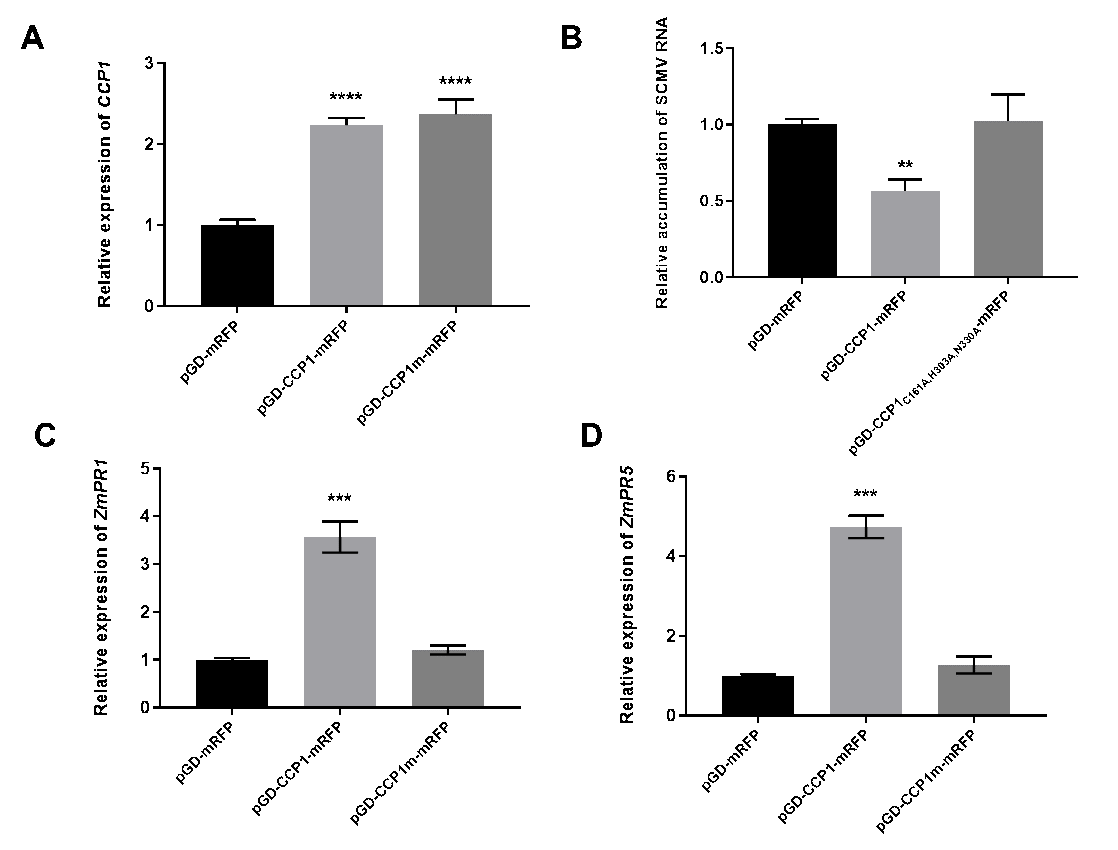

Supplement: S5 Fig — Protoplasts were separately co-transfected with a mixture of pGD-CCP1-mRFP and SCMV RNA, a mixture of pGD-mRFP and SCMV RNA, or a mixture of pGD-CCP1C161A,H303A,N330A-mRFP and SCMV RNA. The expression level of CCP1 (A), the relative accumulation of SCMV RNA (B) and the expression level of SA marker genes ZmPR1 (C) and ZmPR5 (D) were determined at 18 h post transfection. Three independent experiments were conducted with at least three biological replicates per treatment. Error bars are the means ± SE. **, P < 0.01; ***, P < 0.001; ****, P < 0.0001; determined by the unpaired t-test. (TIF) [file ppat.1012086.s006.tif]

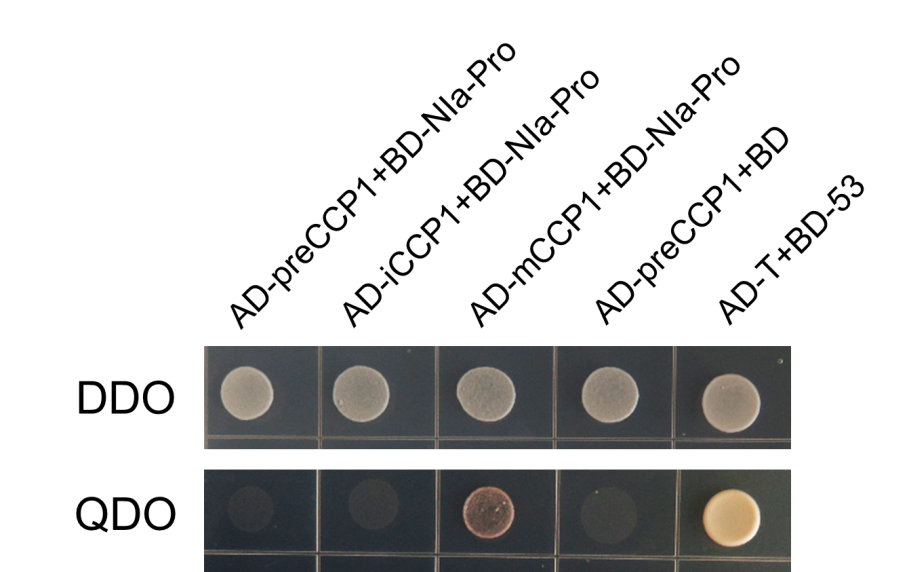

Supplement: S6 Fig — Only mature protease domain of CCP1 (mCCP1) interacts with NIa-Pro, while other forms including precursor of CCP1 (preCCP1) and signal-peptide deletion CCP1 (iCCP1) do not interact with NIa-Pro in yeast cells. Yeast cells grown on the QDO selective medium are the cells with a positive protein–protein interaction. The AD-T+BD-53 serves as the positive control. (TIF) [file ppat.1012086.s007.tif]

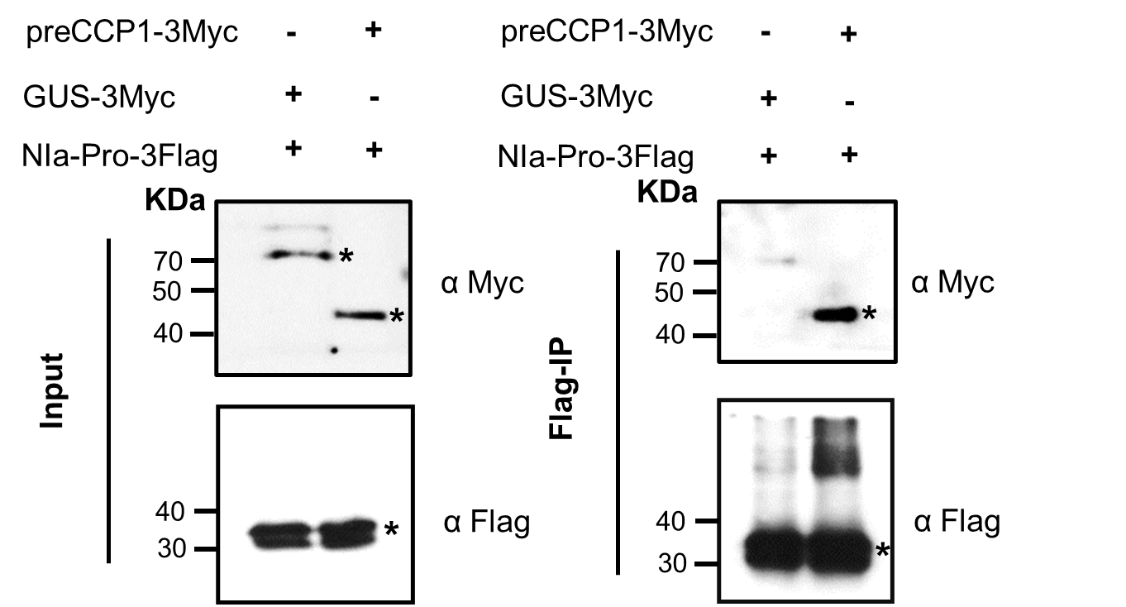

Supplement: S7 Fig — 3Flag-NIa-Pro was co-expressed with preCCP1-3Myc or GUS-3Myc in N. benthamiana leaves through agro-infiltration. Leaf tissues were harvested at 48 hours post agroinfiltration (hpai) for Co-IP analysis. Samples from plants co-expressing 3Flag-NIa-Pro and GUS-3Myc were used as a negative control. Co-IP assay was performed using an anti-Flag affinity agarose gel. Protein samples (Input) and the immunoprecipitated protein samples (Flag-IP) were analyzed through immunoblotting assays using an anti-Flag or an anti-c-Myc antibody. *, the protein bands corresponding to the expected 3Flag-NIa-Pro, GUS-3Myc or preCCP1-3Myc. (TIF) [file ppat.1012086.s008.tif]

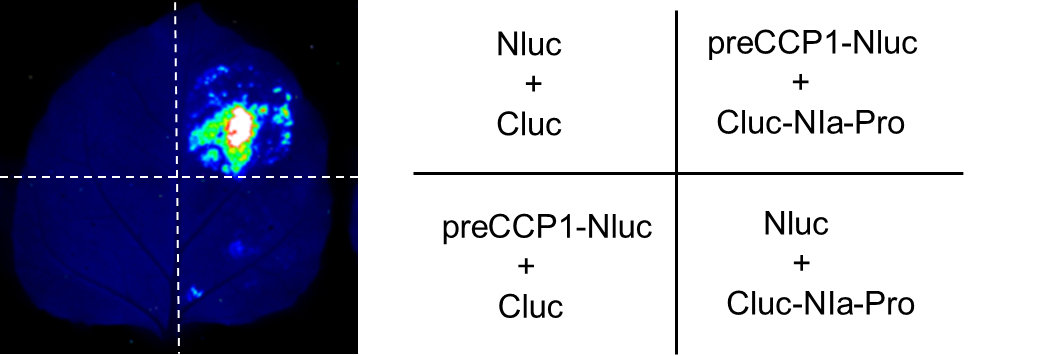

Supplement: S8 Fig — The indicated plasmid pairs were transiently co-expressed in N. benthamiana. Then the luminescent signal was collected at 48 hpai. (TIF) [file ppat.1012086.s009.tif]

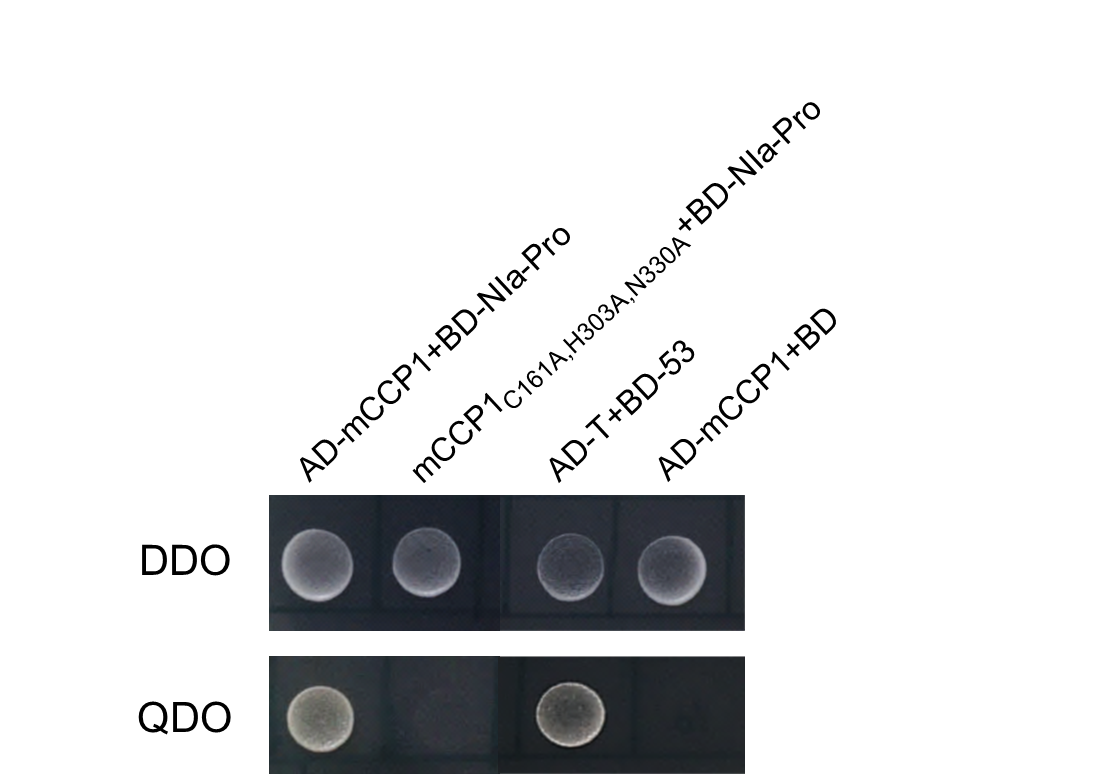

Supplement: S9 Fig — In mCCP1C161A,H303A,N330A, the catalytic triad residues were replaced by Alanine. Yeast cells grown on the QDO selective medium are the cells with a positive protein–protein interaction. The AD-T+BD-53 serves as the positive control. (TIF) [file ppat.1012086.s010.tif]

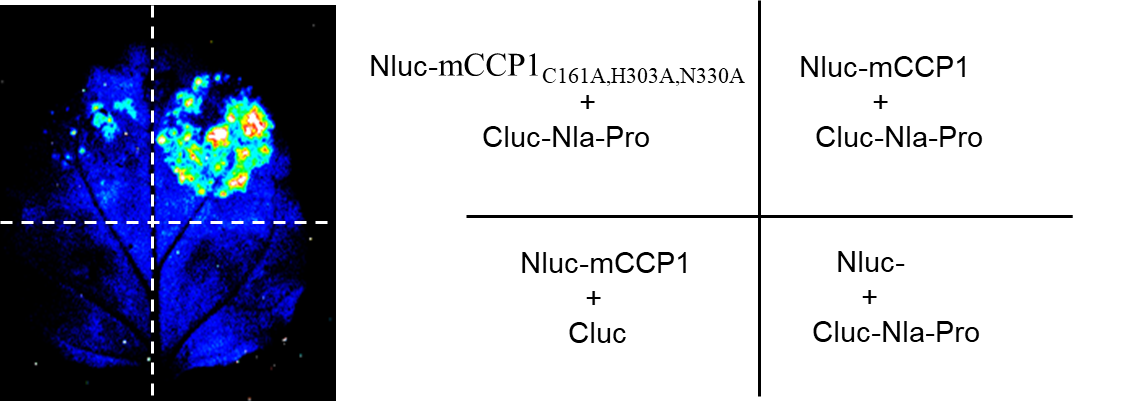

Supplement: S10 Fig — In mCCP1C161A,H303A,N330A, the catalytic triad residues were replaced by Alanine. The indicated plasmid pairs were transiently co-expressed in N. benthamiana. Then the luminescent signal was collected at 48 hpai. (TIF) [file ppat.1012086.s011.tif]

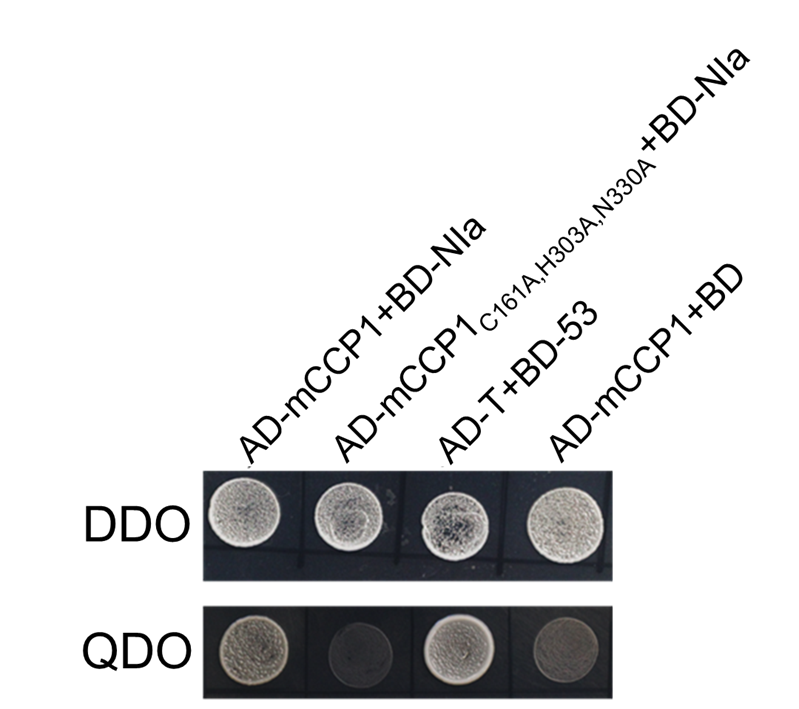

Supplement: S11 Fig — The plasmids pGADT7-mCCP1 and pGBKT7-NIa, or pGADT7-mCCP1C161A, H303A, N330A and pGBKT7-NIa were co-transformed into yeast. The co-transformants were grown on the selective medium at 30°C for 3–4 d. Yeast cells grown on the QDO selective medium are the cells with a positive protein–protein interaction. The AD-T+BD-53 serves as the positive control. (TIF) [file ppat.1012086.s012.tif]

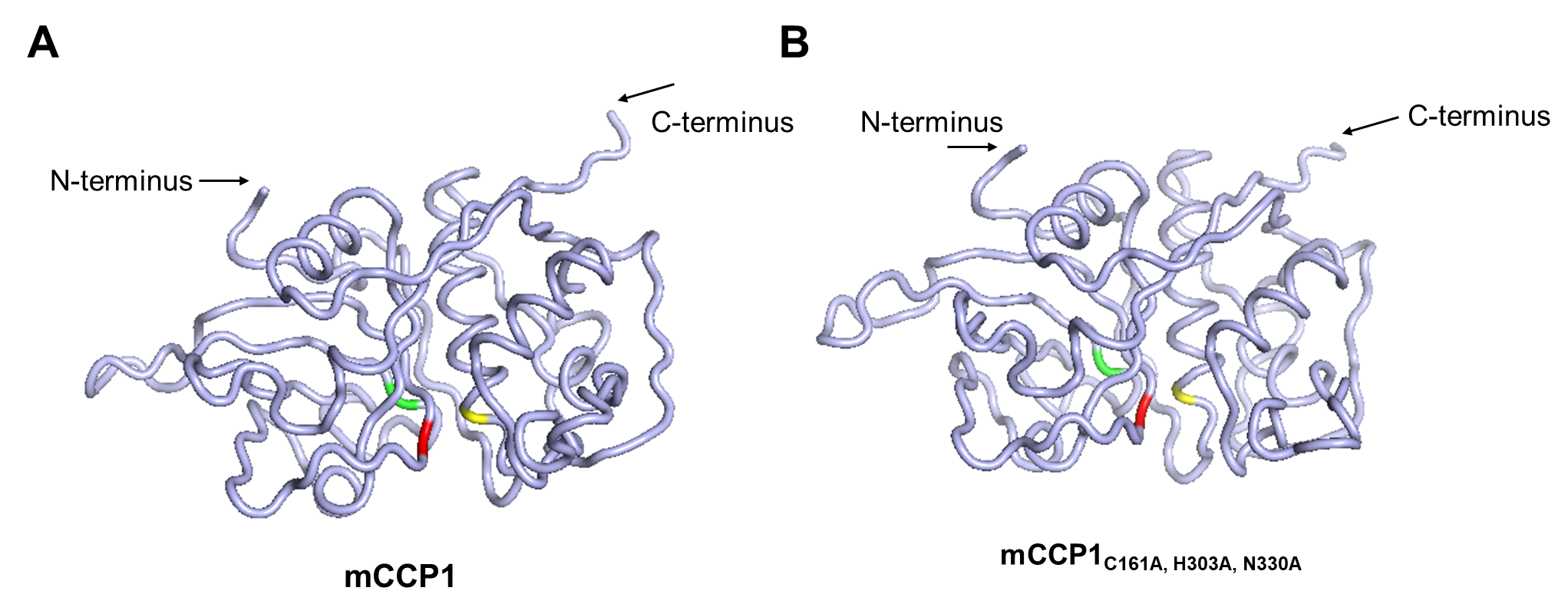

Supplement: S12 Fig — The N and C termini of the protein are indicated by black arrows, and the catalytic triad key residues are labeled as yellow (C161), red (H303), and green (N330). (TIF) [file ppat.1012086.s013.tif]

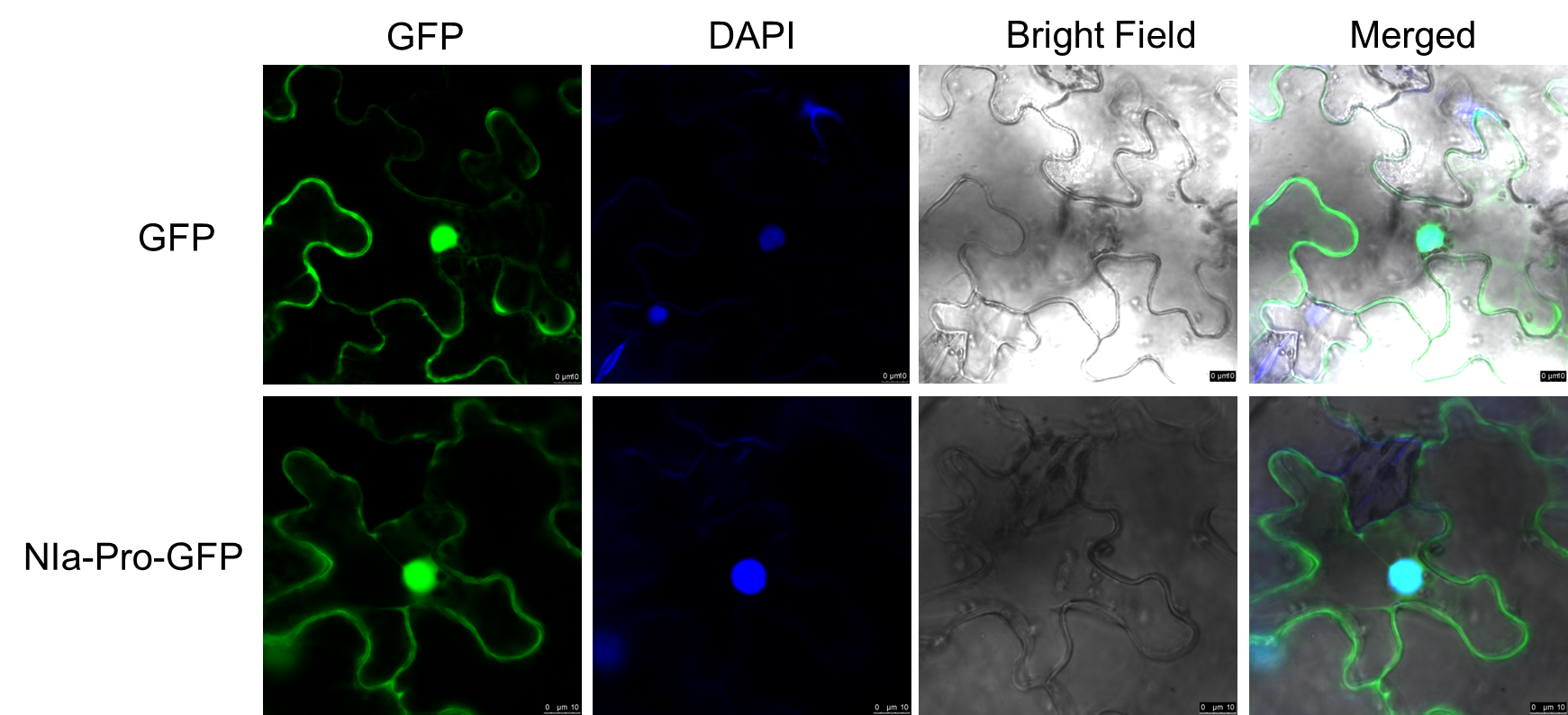

Supplement: S13 Fig — Confocal images of N. benthamiana leaf cells expressing NIa-Pro-GFP or GFP at 48 hpai. DAPI staining indicates the nucleus of tobacco cells, revealing that the green fluorescence of NIa-Pro-GFP is localized not only in the cytoplasm but also in the nucleus. Images (left to right) showed GFP fluorescence, DAPI fluorescence, bright field and overlay of the three images. Scale bars, 10 μm. (TIF) [file ppat.1012086.s014.tif]

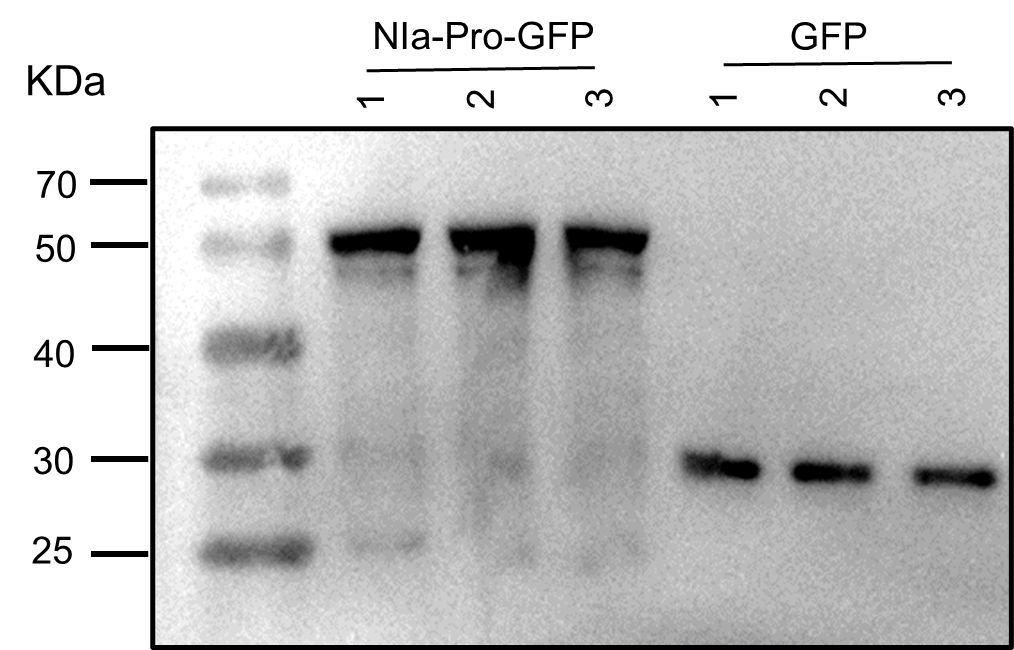

Supplement: S14 Fig — At 48 hpai, infiltrated leaves were sampled for immunoblotting assays using an anti-GFP antibody. (TIF) [file ppat.1012086.s015.tif]

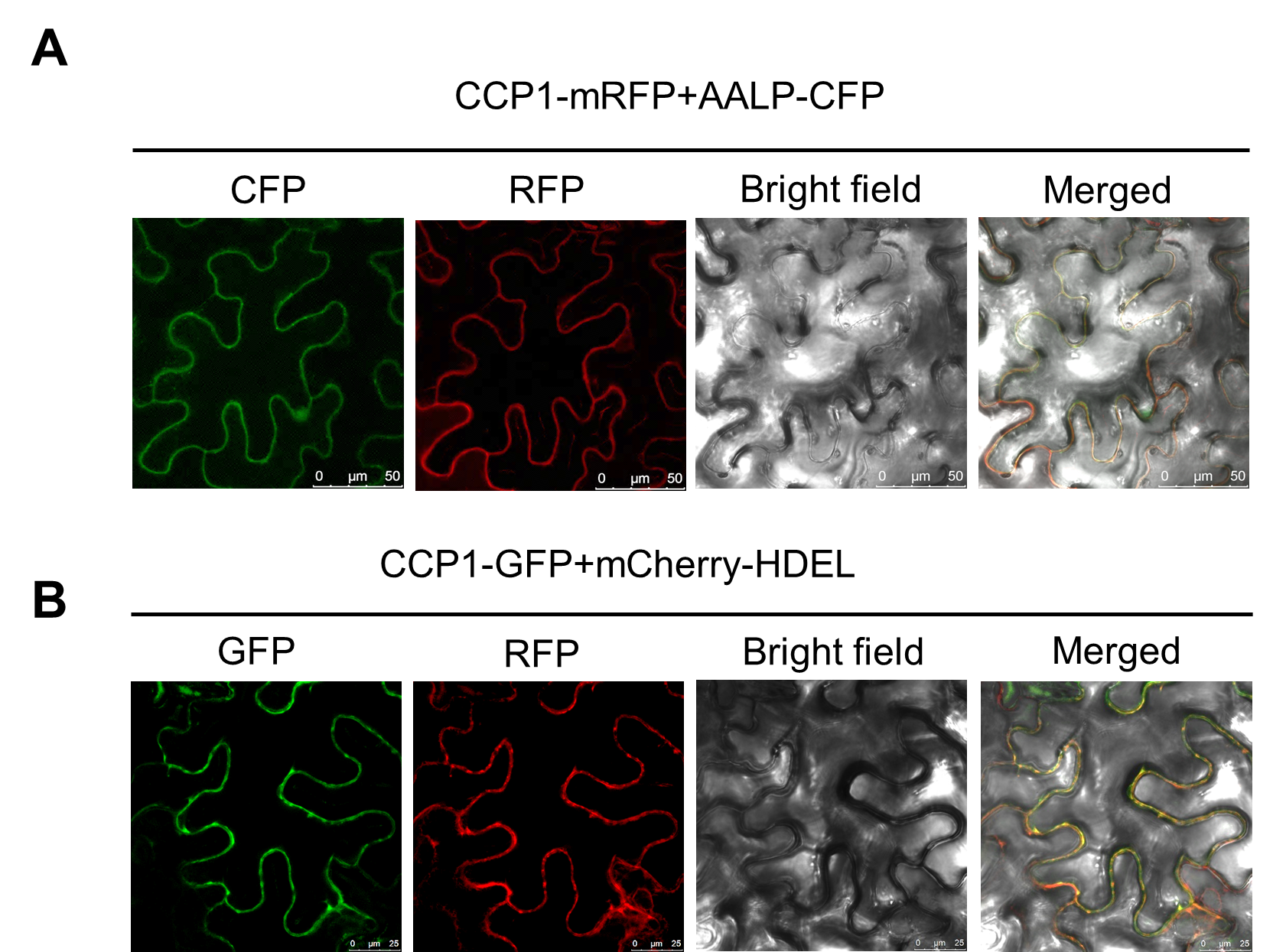

Supplement: S15 Fig — CCP1 colocalizes with the lytic vacuolar marker AALP (A) and the endoplasmic reticulum marker HDEL (B) in the epidermal cells of N. benthamiana leaf. Images (left to right) showed CFP or GFP fluorescence, RFP fluorescence, bright field and overlay of the three images. Scale bars, 50 μm. (TIF) [file ppat.1012086.s016.tif]

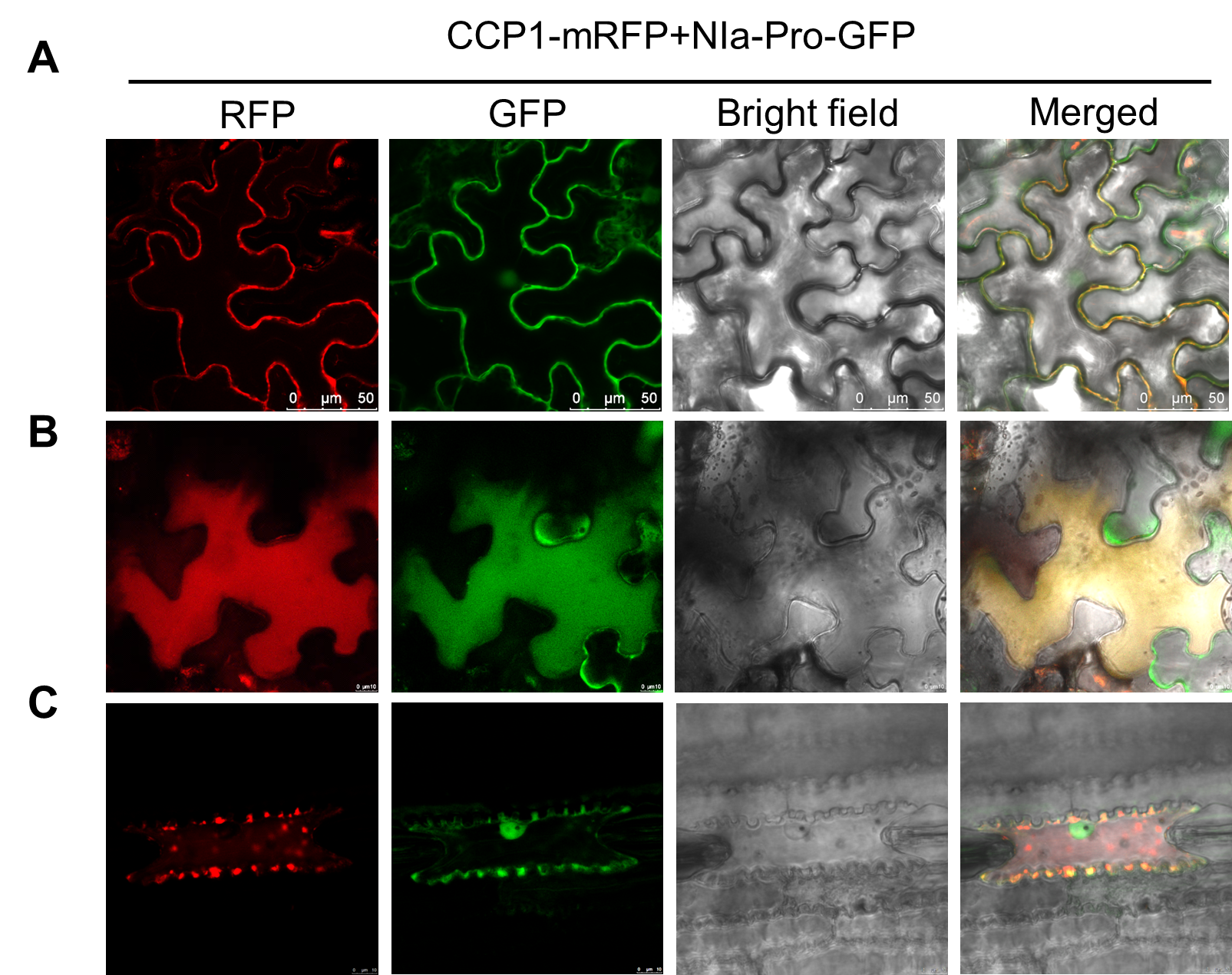

Supplement: S16 Fig — Confocal images of N. benthamiana leave cells co-expressing CCP1-mRFP and NIa-Pro-GFP at 48 (A) and 72 hpai (B). C) The overlay of CCP1-mRFP and NIa-Pro-GFP in the cytoplasm of maize cell at 16 h after bombardment. Images (left to right) showed RFP fluorescence, GFP fluorescence, bright field and overlay of the three images. Scale bars, 50 μm. (TIF) [file ppat.1012086.s017.tif]

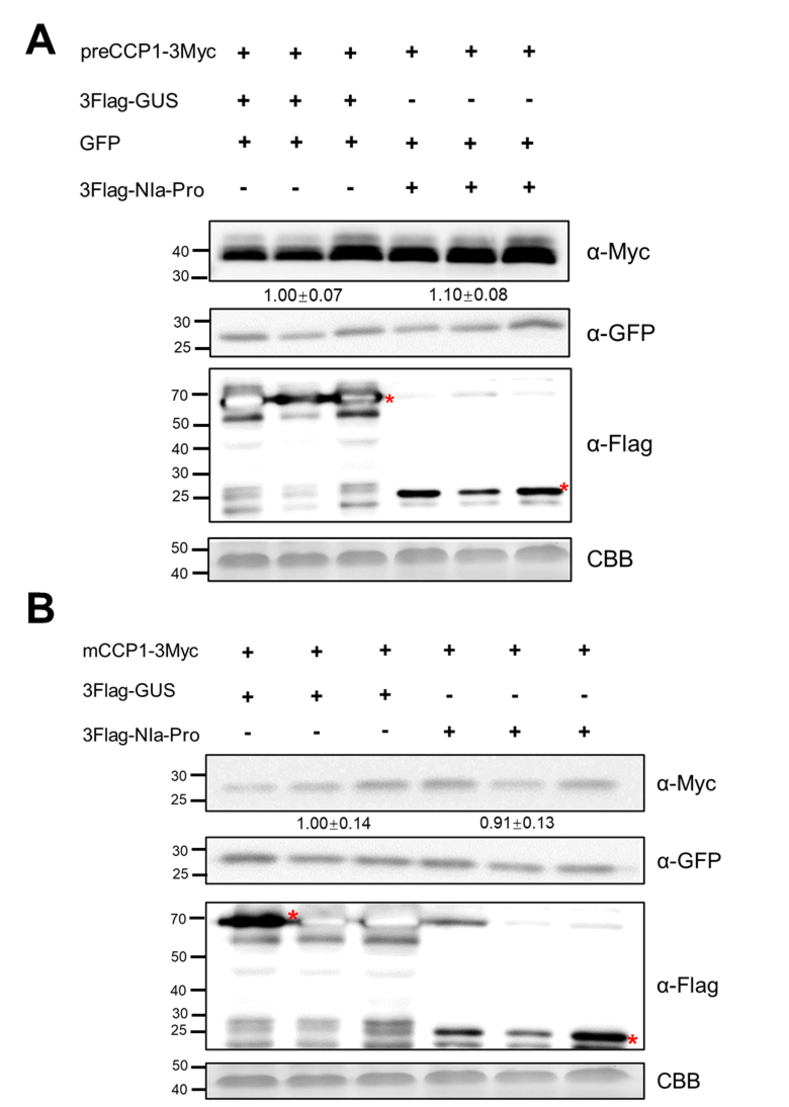

Supplement: S17 Fig — pGD-preCCP1-3Myc or pGD-mCCP1-3Myc was co-infiltrated with pGD-GFP plus pGD-3Flag-NIa-Pro or pGD-3Flag-GUS (control) in N. benthamiana leaves. At 3 dpi, infiltrated leaves were sampled and analyzed for the accumulation levels of preCCP1-3Myc (A) and mCCP1-3Myc (B) via immunoblotting assays. The strength of detection signal was analyzed using the ImageJ software. The red asterisk indicates the protein band corresponding to preCCP1- or mCCP1-3Myc. Three independent experiments were conducted with at least three biological replicates per treatment. (TIF) [file ppat.1012086.s018.tif]

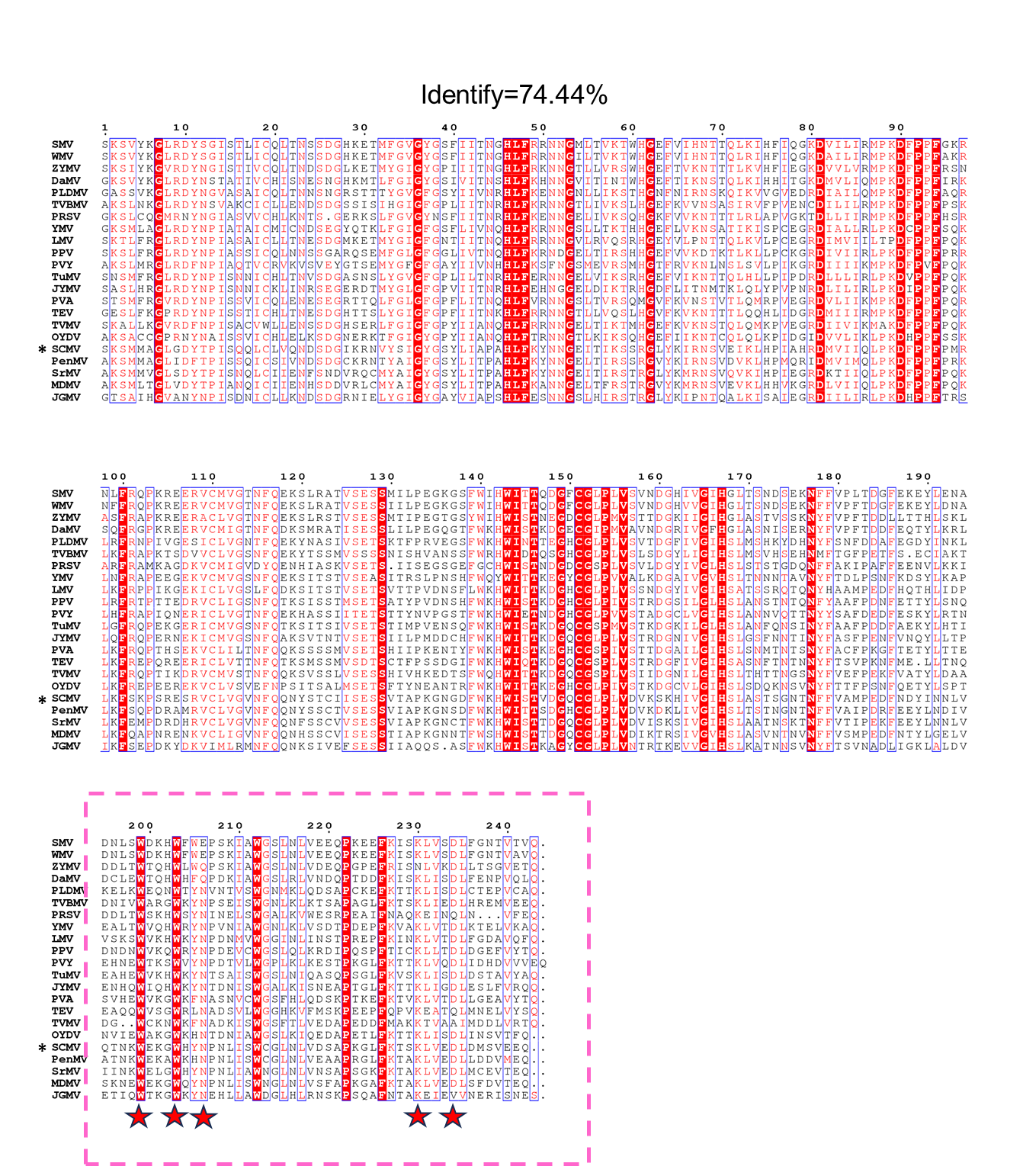

Supplement: S18 Fig — The following viruses were included in the analysis: soybean mosaic virus (SMV, AAB22819.2), watermelon mosaic virus (WMV, AAA48497.2), dasheen mosaic virus (DaMV, NP_613274.1), papaya leaf distortion mosaic virus (PLDMV, NP_870995.1), papaya ringspot virus (PRSV, AAG47346.1), yam mosaic virus (YMV, QGA88722.1), lettuce mosaic virus (LMV, QEG79196.1), plum pox virus (PPV, CVK35891.1), potato virus Y (PVY, AKG94974.1), turnip mosaic virus (TuMV, QBQ58061.1), japanese yam mosaic virus (JYMV, NP_051161.1), potato virus A (PVA, YP_006395324.1), tobacco etch virus (TEV, ABJ16044.1), tobacco vein mottling virus (TVMV, CAA27720.1), onion yellow dwarf virus (OYDV, NP_871002.1), sugarcane mosaic virus (SCMV, AMM72620.1), pennisetum mosaic virus (PenMV, YP_006395348.1), sorghum mosaic virus (SrMV, CAX36858.1), maize dwarf mosaic virus (MDMV, NP_569138.1) and johnsongrass mosaic virus (JGMV, ALS88434.1). The regions highlighted by the pink bounding box are hypothesized to represent the functional peptide of the NIa-Pro C-terminus. The amino acid residues marked by pentagram symbols represent the selected functional amino acids for mutagenesis. (TIF) [file ppat.1012086.s019.tif]

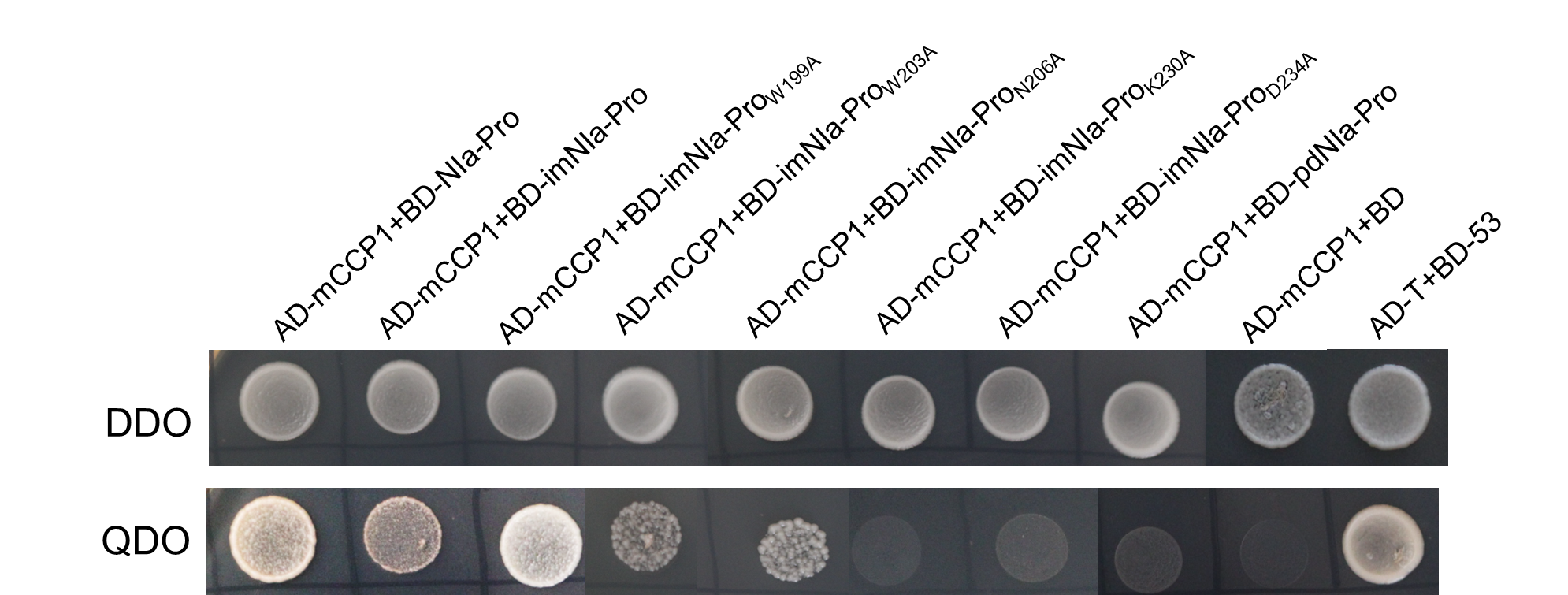

Supplement: S19 Fig — Yeast cells grown on the QDO selective medium are the cells with a positive protein–protein interaction. The AD-T+BD-53 serves as the positive control. (TIF) [file ppat.1012086.s020.tif]

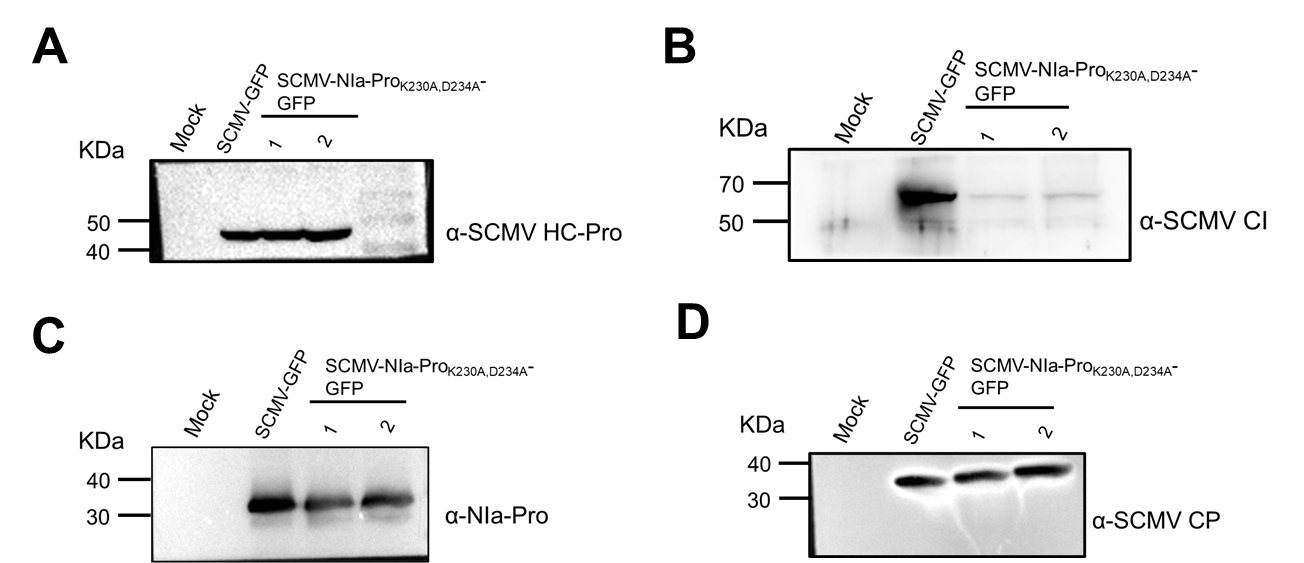

Supplement: S20 Fig — At 7 dpi, leaf samples were collected for immunoblot analysis using specific antibodies to detect the expression of SCMV HC-Pro (A), CI (B), NIa-Pro (C), and CP (D). (TIF) [file ppat.1012086.s021.tif]

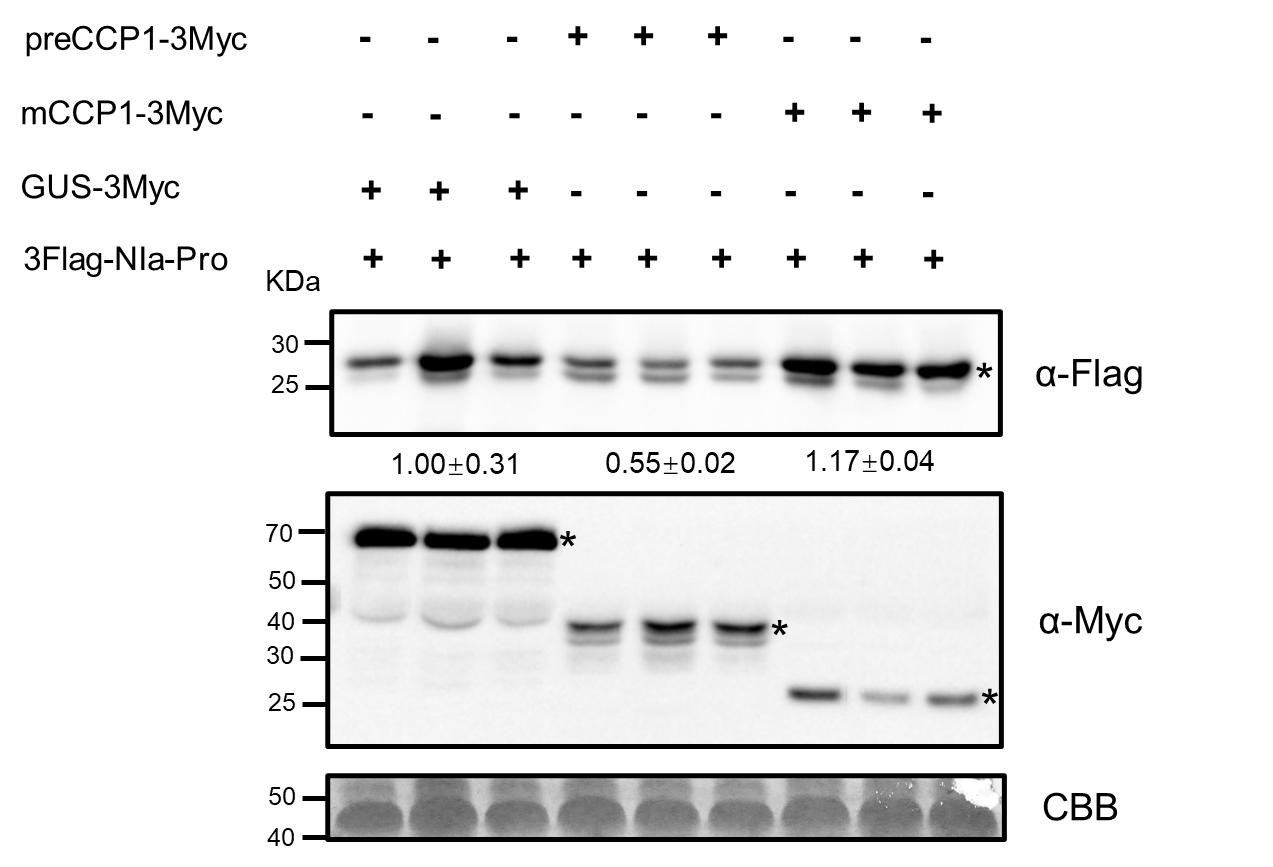

Supplement: S21 Fig — pGD-preCCP1-3Myc or pGD-mCCP1-3Myc was co-infiltrated with pGD-3Flag-NIa-Pro in N. benthamiana leaves. pGD-GUS-3Myc co-expressed with pGD-3Flag-NIa-Pro was used as control. At 3 dpi, infiltrated leaves were sampled and analyzed for the accumulation levels of 3 Flag-NIa-Pro via immunoblotting assays. The strength of detection signal was analyzed using the ImageJ software. Three independent experiments were conducted with at least three biological replicates per treatment. (TIF) [file ppat.1012086.s022.tif]
