# Supplementary material for: NIa-Pro of sugarcane mosaic virus targets Corn Cysteine Protease 1 (CCP1) to undermine salicylic acid-mediated defense in maize
Source: PLoS Pathog. 2024 Mar 14;20(3):e1012086. doi: 10.1371/journal.ppat.1012086 (PMC10965072; doi:10.1371/journal.ppat.1012086)
Supplement: S1 Table — (DOCX) [file ppat.1012086.s023.docx]

**Supplementary information-table**

**Table S1.** The expression of *PLCP* genes was modulated by diverse plant viruses.

| Virus | Genus | Host | PLCPs (Up-regulate) | PLCPs (Down-regulate) | Reference |
| --- | --- | --- | --- | --- | --- |
| plum pox virus | *Potyvirus* | *Nicotiana benthamiana* | *Cathepsin B* |  | [1] |
| tobacco etch virus | *Potyvirus* | *Arabidopsis thaliana* | *AALP*, *Cathepsin B* | *RD21A*，*XCP1*，*XCP2* | [2] |
| potato virus Y | *Potyvirus* | *Solanum tuberosum* L. | *XCP2* | *RCR3* | [3] |
| potato virus X | *Potexvirus* | *N. benthamiana* | *Cathepsin B* |  | [4] |
| cucumber mosaic virus | *Cucumovirus* | *Lilium regale* | *SAG39* | *Cathepsin B*, *CEP1* | [5] |
| mungbean yellow mosaic India virus | *Begomovirus* | *Glycine max* | *GmMir1*, *GmALP* |  | [6] |

**References:**

1. Dardick C. Comparative expression profiling of *Nicotiana benthamiana* leaves systemically infected with three fruit tree viruses. Mol Plant Microbe In. 2007;20(8):1004–17. doi: 10.1094/Mpmi-20-8-1004. PubMed PMID: WOS:000248233000012.
2. Hillung J, Cuevas JM, Elena SF. Transcript profiling of different *Arabidopsis thaliana* ecotypes in response to tobacco etch potyvirus infection. Front Microbiol. 2012;3. doi: ARTN 229 10.3389/fmicb.2012.00229. PubMed PMID: WOS:000208863600277.
3. Goyer A, Hamlin L, Crosslin JM, Buchanan A, Chang JH. RNA-Seq analysis of resistant and susceptible potato varieties during the early stages of potato virus Y infection. Bmc Genomics. 2015;16. doi: ARTN 472 10.1186/s12864-015-1666-2. PubMed PMID: WOS:000356479100002.
4. Pacheco R, Garcia-Marcos A, Manzano A, de Lacoba MG, Camanes G, Garcia-Agustin P, et al. Comparative analysis of transcriptomic and hormonal responses to compatible and incompatible plant-virus interactions that lead to cell death. Mol Plant Microbe In. 2012;25(5):709–23. doi: 10.1094/Mpmi-11-11-0305. PubMed PMID: WOS:000303083000011.
5. Sun DY, Zhang XG, Zhang QY, Ji XT, Jia Y, Wang H, et al. Comparative transcriptome profiling uncovers a Lilium regale NAC transcription factor, LrNAC35, contributing to defence response against cucumber mosaic virus and tobacco mosaic virus. Mol Plant Pathol. 2019;20(12):1662–81. doi: 10.1111/mpp.12868. PubMed PMID: WOS:000487883900001.
6. Yadav RK, Chattopadhyay D. Differential soybean gene expression during early phase of infection with mungbean yellow mosaic India virus. Mol Biol Rep. 2014;41(8):5123–34. doi: 10.1007/s11033-014-3378-0. PubMed PMID: WOS:000339910900027.
