# Supplementary material for: NIa-Pro of sugarcane mosaic virus targets Corn Cysteine Protease 1 (CCP1) to undermine salicylic acid-mediated defense in maize
Source: PLoS Pathog. 2024 Mar 14;20(3):e1012086. doi: 10.1371/journal.ppat.1012086 (PMC10965072; doi:10.1371/journal.ppat.1012086)
Supplement: S2 Table — (DOCX) [file ppat.1012086.s024.docx]

**Supplementary information-table**

**Table S2.** Primers used in this study.

| Name | Sequence (5'-3') |
| --- | --- |
| CMV-CCP1-silencing-1F | ATGTCCGAGTCTGAGTCTCTCGCGTCGGCCGCC |
| CMV-CCP1-silencing-1R | AAGGGGAGGTTCTAGCGCGCGGCGCAGGTTGGC |
| AD-preCCP1-F | GAGGCCAGTGAATTCATGGCTCATCGCGTTCTCCTC |
| AD-CCP1-R | GAGCTCGATGGATCCCTACTCCTTCGAGGCGTGGA |
| AD-iCCP1-F | GAGGCCAGTGAATTCGCCGCCGTCGACGCGGAGGAC |
| AD-mCCP1-F | GAGGCCAGTGAATTCCTCCCCGACGATTTCGACTGG |
| CysM-CCP1-2F | GCGGGTCGGCTTGGTCGTTCAGCG |
| CysM-CCP1-2R | AACGACCAAGCCGACCCGCACGAA |
| HisM-CCP1-2F | ACCTTGACGCTGGTGTTCTCCTTG |
| HisM-CCP1-2R | AGAACACCAGCGTCAAGGTGCCTG |
| AsnM-CCP1-2F | TCATCAAGGCTTCGTGGGGCGAGA |
| AsnM-CCP1-2R | CCCCACGAAGCCTTGATGATCCAG |
| mCCP1-3Flag-F | AATTCTGCAGTCGACGATGCTCCCCGACGATTTCGACT |
| CCP1-3Flag-R | AGATCCGGTGGATCCCTACTCCTTCGAGGCGTGGAC |
| mCCP1-3Myc-F | AATTCTGCAGTCGACATGGCCTCCTCCGAGGACGTCATC |
| CCP1-3Myc-R | AGATCCGGTGGATCCTTAAAGATCCTCCTCAGAAAT |
| pGD-Flag-SC-NIa-Pro-F | AATTCTGCAGTCGACGATGTCGAAATCGATGATGGCA |
| pGD-Flag-SC-NIa-Pro -R | AGATCCGGTGGATCCTCATTGTTCTTCAACGCTCATATC |
| Nluc-mCCP1-F | GACGAGCTCGGTACCATGCTCCCCGACGATTTCGACT |
| Nluc-preCCP1-R | CGAGATCTGGTCGACCTCCTTCGAGGCGTGGACTGC |
| Cluc-SCMV-NIa-Pro-F | TCCCGGGGCGGTACCATGTCGAAATCGATGATGGCAGGG |
| Cluc-SCMV-NIa-Pro-R | GCTCTGCAGGTCGACTCATTGTTCTTCAACGCTCATATCA |
| pColdTF-mCCP1-F | ATGGAGCTCGGTACCCTCCCCGACGATTTCGACTGG |
| pColdTF-CCP1-R | GACAAGCTTGAATTCCTACTCCTTCGAGGCGTGGAC |
| pGEX-SCMV-NIa-Pro- F | GTATTTTCAGGGATCCATGTCGAAATCGATGATGGCAG |
| pGEX-SCMV-NIa-Pro- R | TGGTGGTGGTGCTCGAGTTGTTCTTCAACGCTCATATC |
| FoMV-NIa-Pro-F | AGGACACAAGGGCAACAGCGGCCGCATGGACTACAA |
| FoMV-NIa-Pro-R | CAATGCGGTCGTTGAGTGTCTAGATCATTGTTCTTCAACG |
| **RT-PCR primers** |  |
| ZmUbi-qRT-F | GGAAAAACCATAACCCTGGA |
| ZmUbi-qRT-R | ATATGGAGAGAGGGCACCAG |
| SCMV-CP-qRT-F | GGCGAGACTCAGGAGAATACA |
| SCMV-CP-qRT-R | ACACGCTACACCAGAAGACACT |
| ZmPR1-qRT-F | GGCGAGAGCCCCTACTAGAC |
| ZmPR1-qRT-R | AAATCGCCTGCATGGTTTTA |
| ZmPR5-qRT-F | GTCATCGACGGCTACAACCT |
| ZmPR5-qRT-R | CACGGGCAGAAGGTGACT |
| Oligod(T) | TTTTTTTTTTTTTTTTTT |
| CCP1-qRT-F | CATCTGTGGCAGGCACCTTGAC |
| CCP1-qRT-R | TGGACTGCGGACACGGTAGAGA |
