# Supplementary material for: NIa-Pro of sugarcane mosaic virus targets Corn Cysteine Protease 1 (CCP1) to undermine salicylic acid-mediated defense in maize
Source: PLoS Pathog. 2024 Mar 14;20(3):e1012086. doi: 10.1371/journal.ppat.1012086 (PMC10965072; doi:10.1371/journal.ppat.1012086)
Supplement: S1 Information — (PPTX) [file ppat.1012086.s025.pptx]

## Slide 1
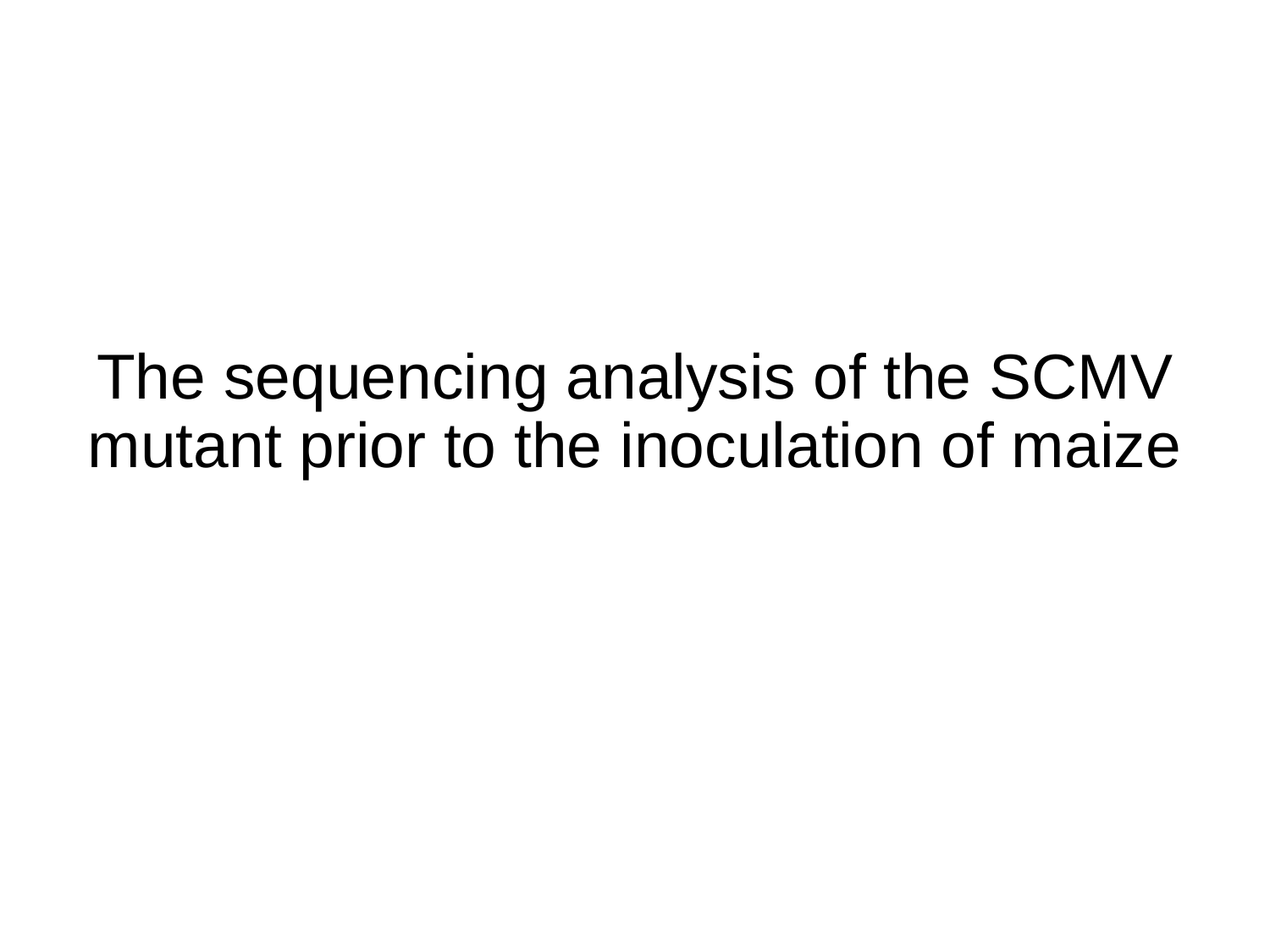

# The sequencing analysis of the SCMV mutant prior to the inoculation of maize

## Slide 2
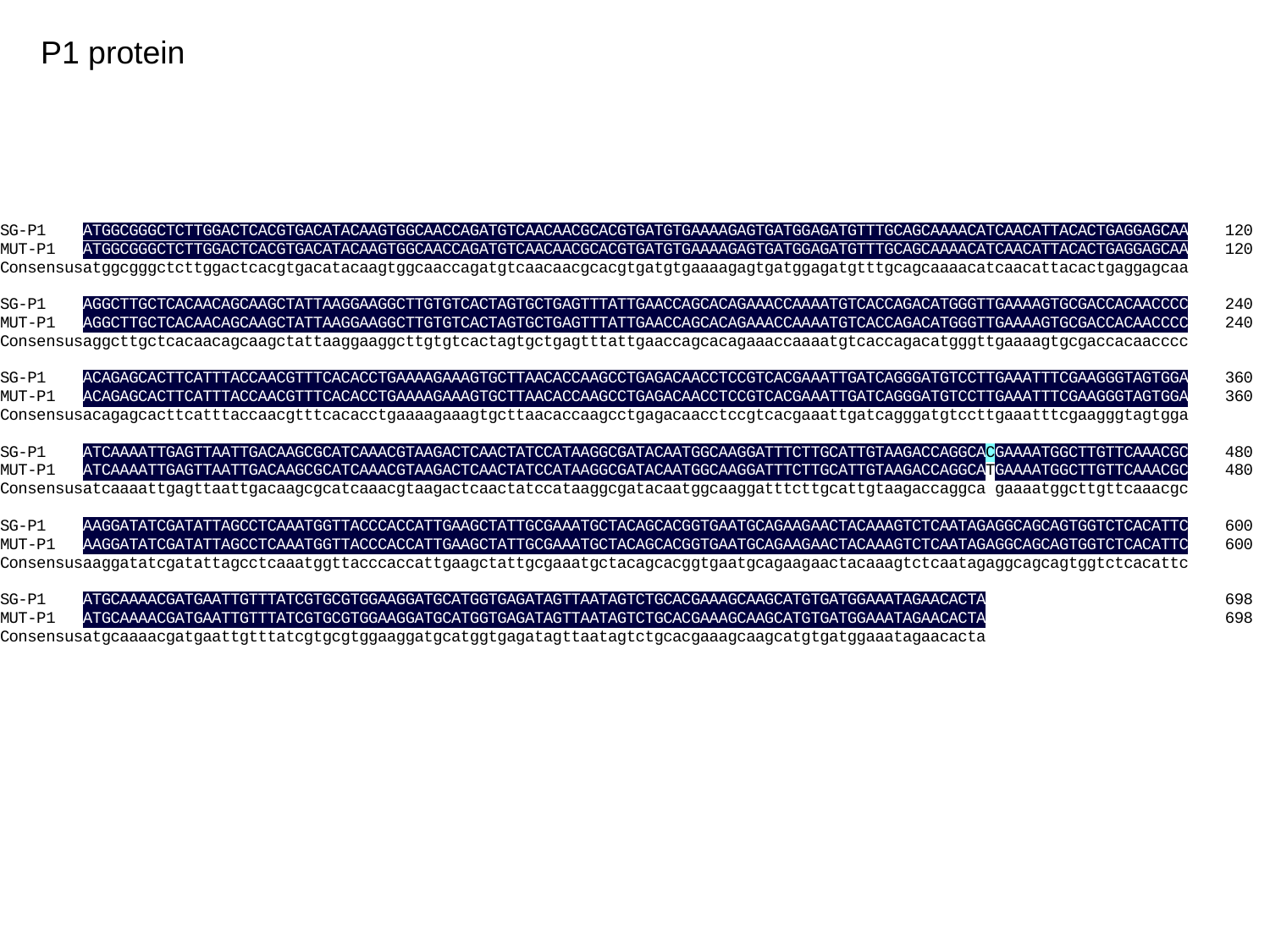

P1 protein

## Slide 3
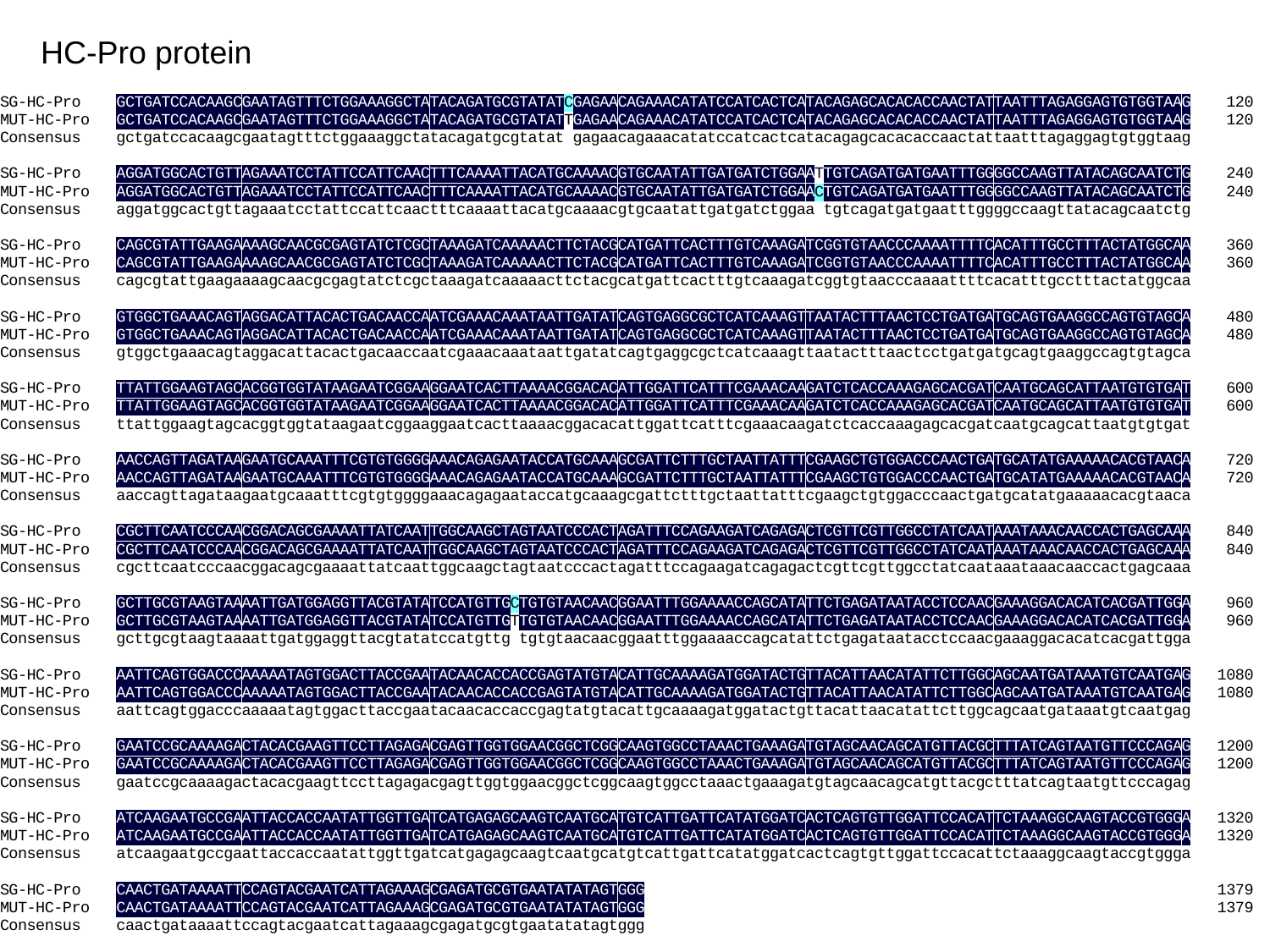

HC-Pro protein

## Slide 4
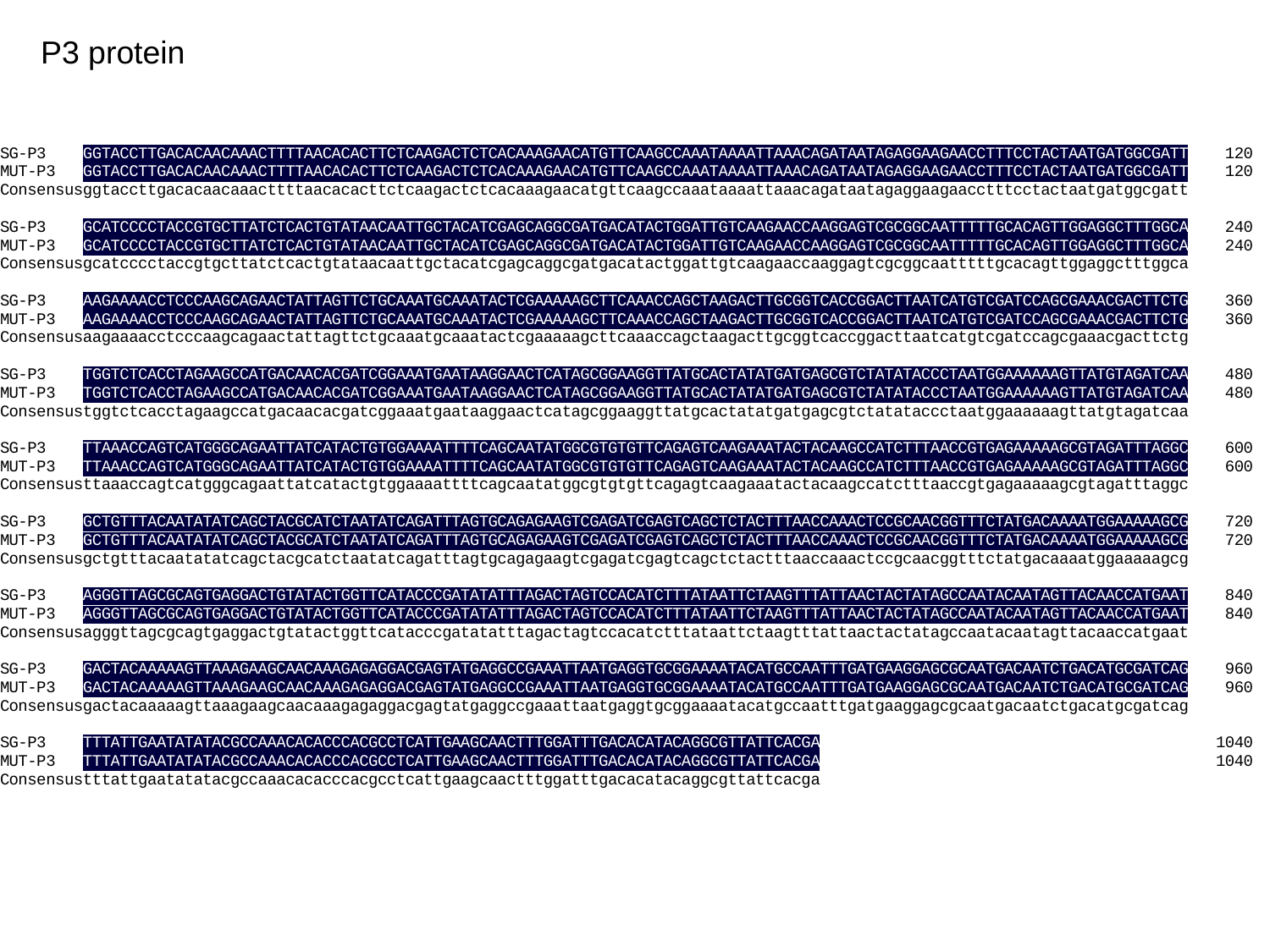

P3 protein

## Slide 5
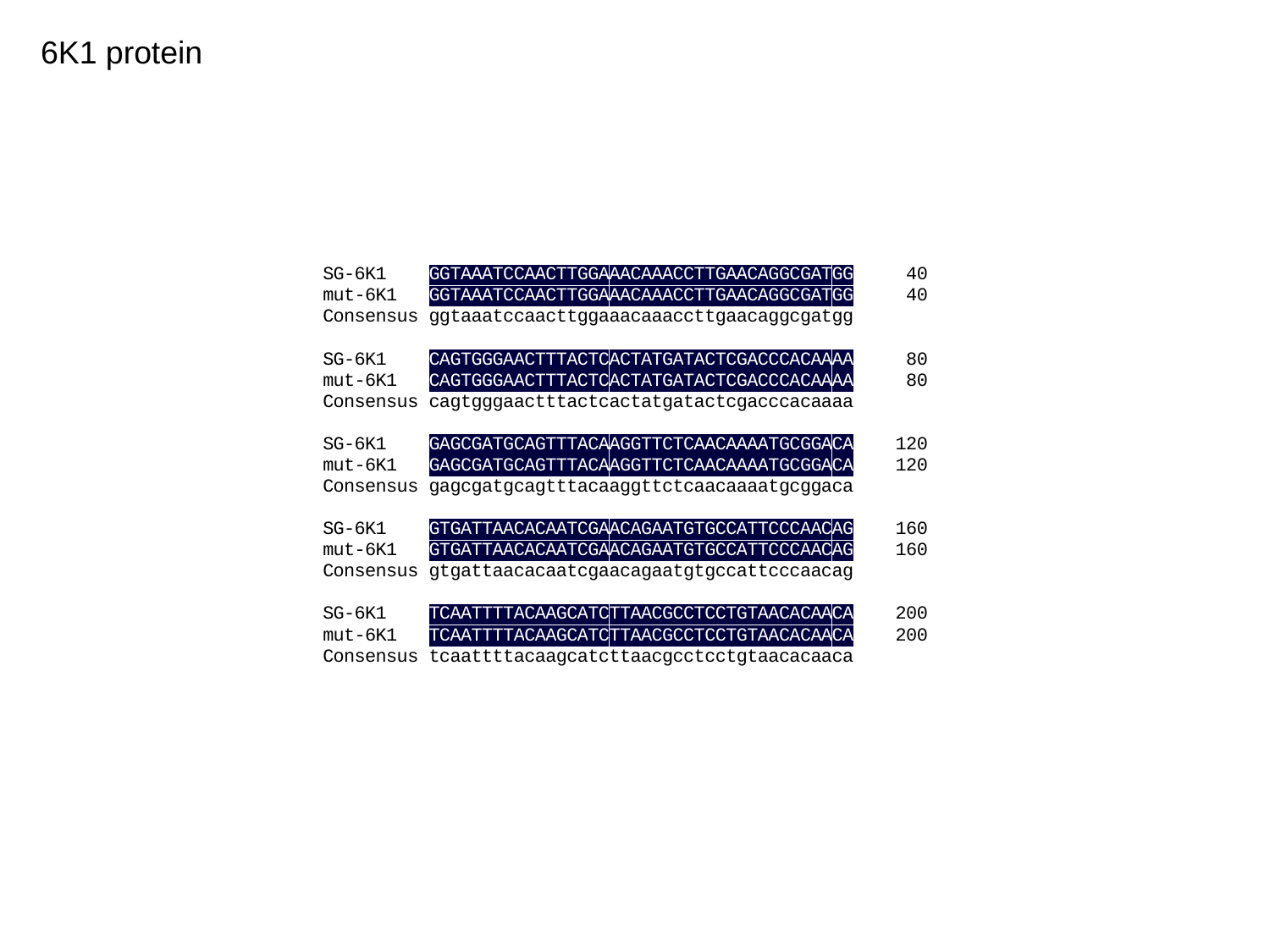

6K1 protein

## Slide 6
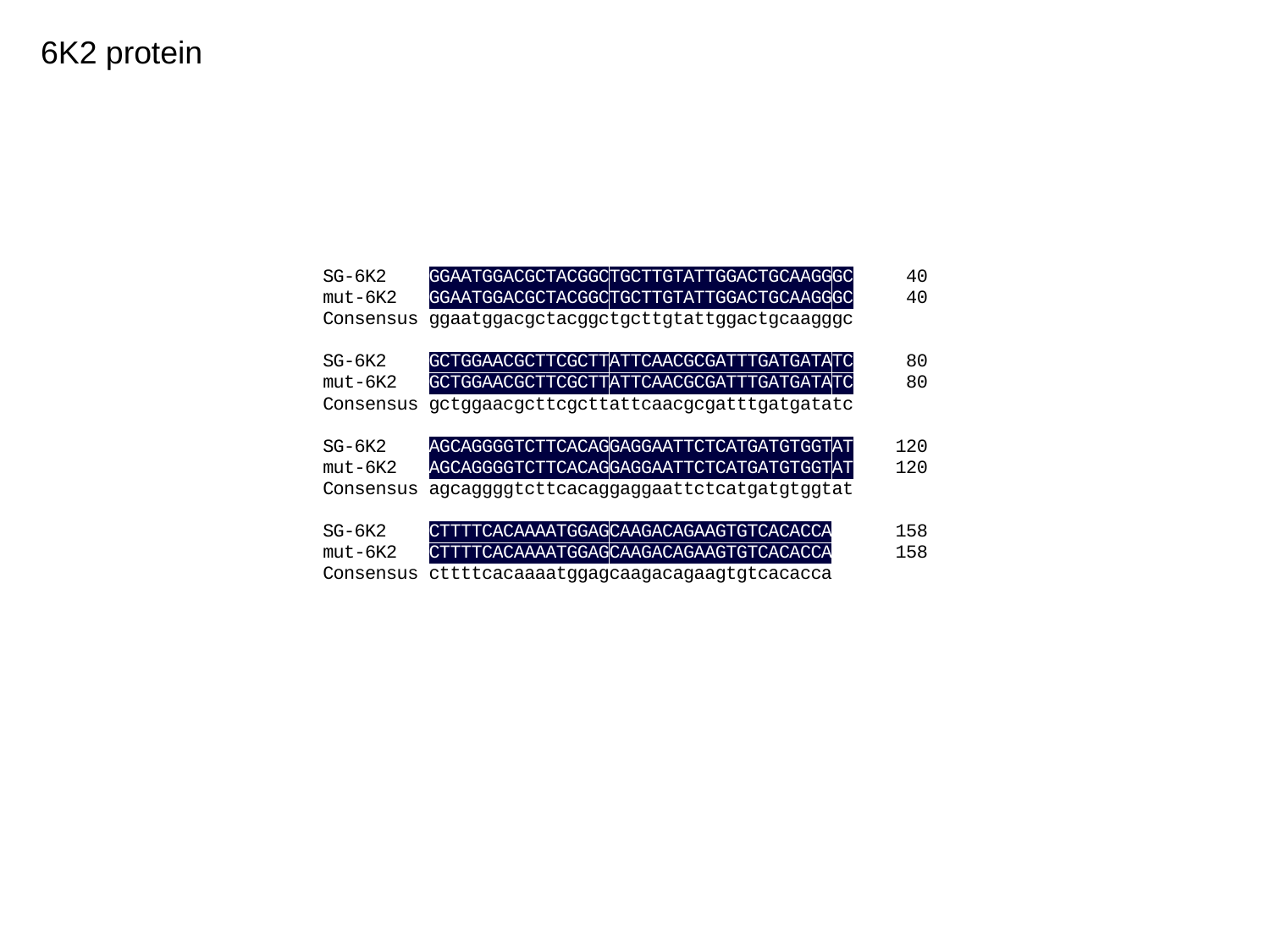

6K2 protein

## Slide 7
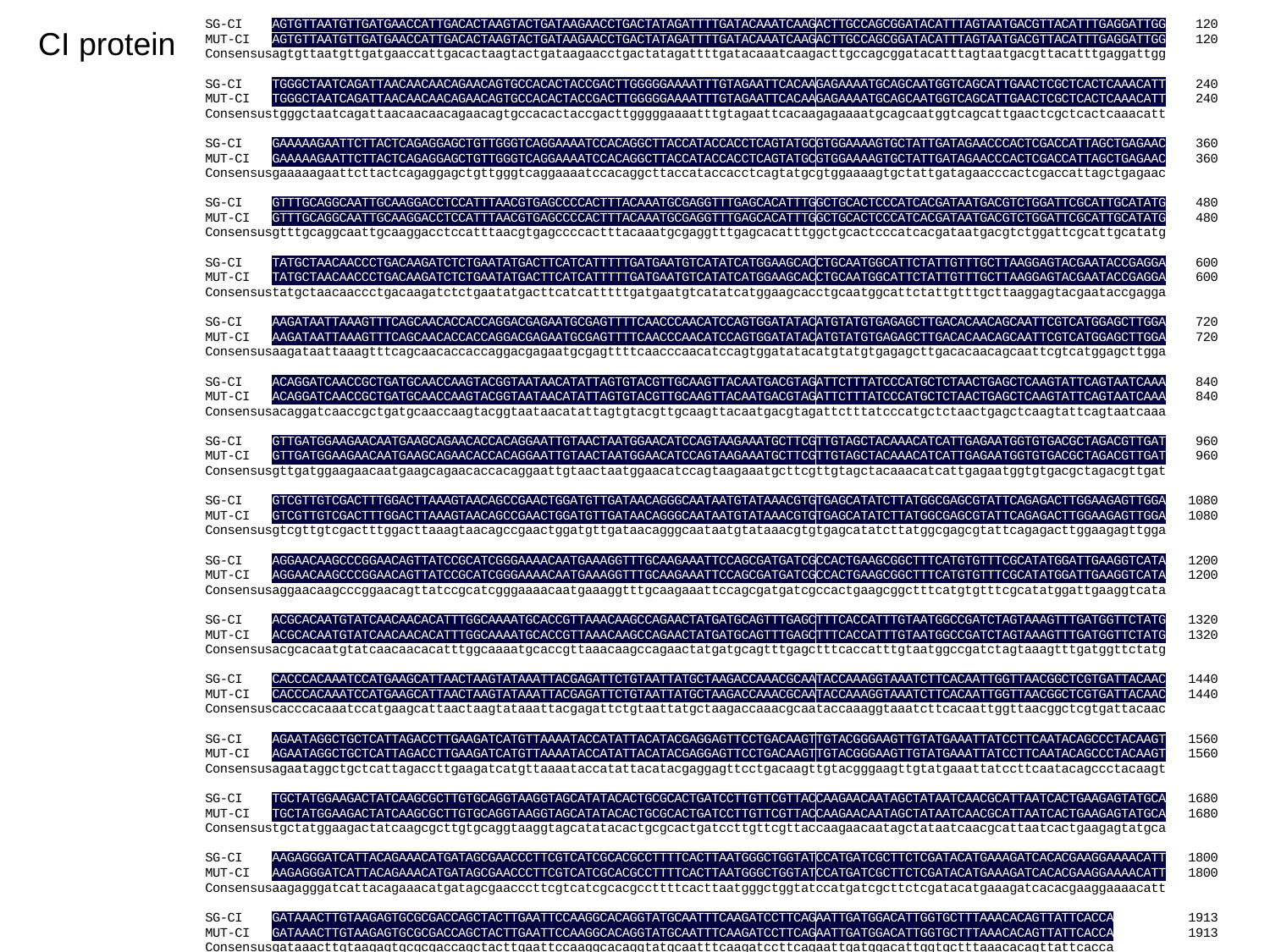

CI protein

## Slide 8
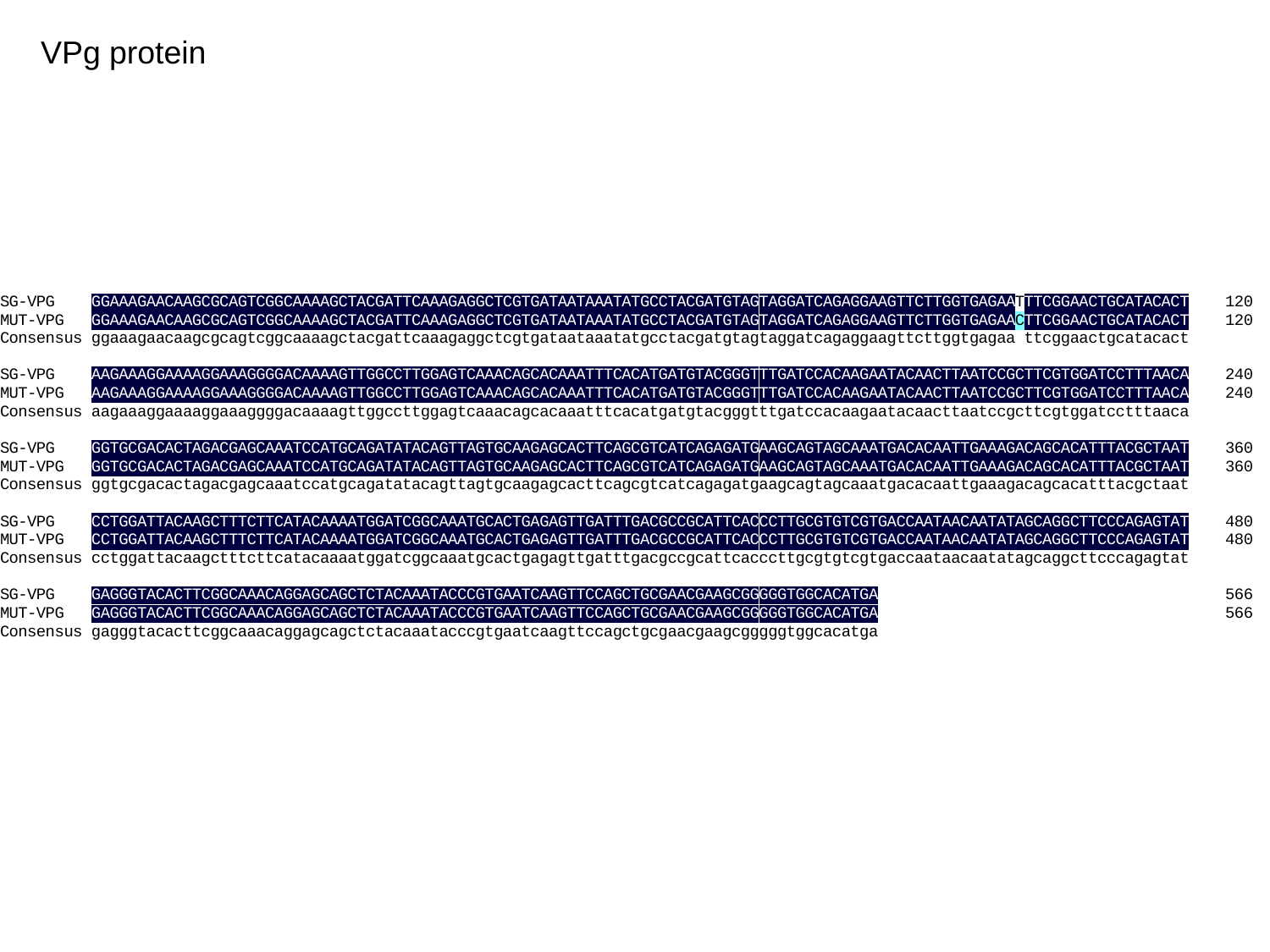

VPg protein

## Slide 9
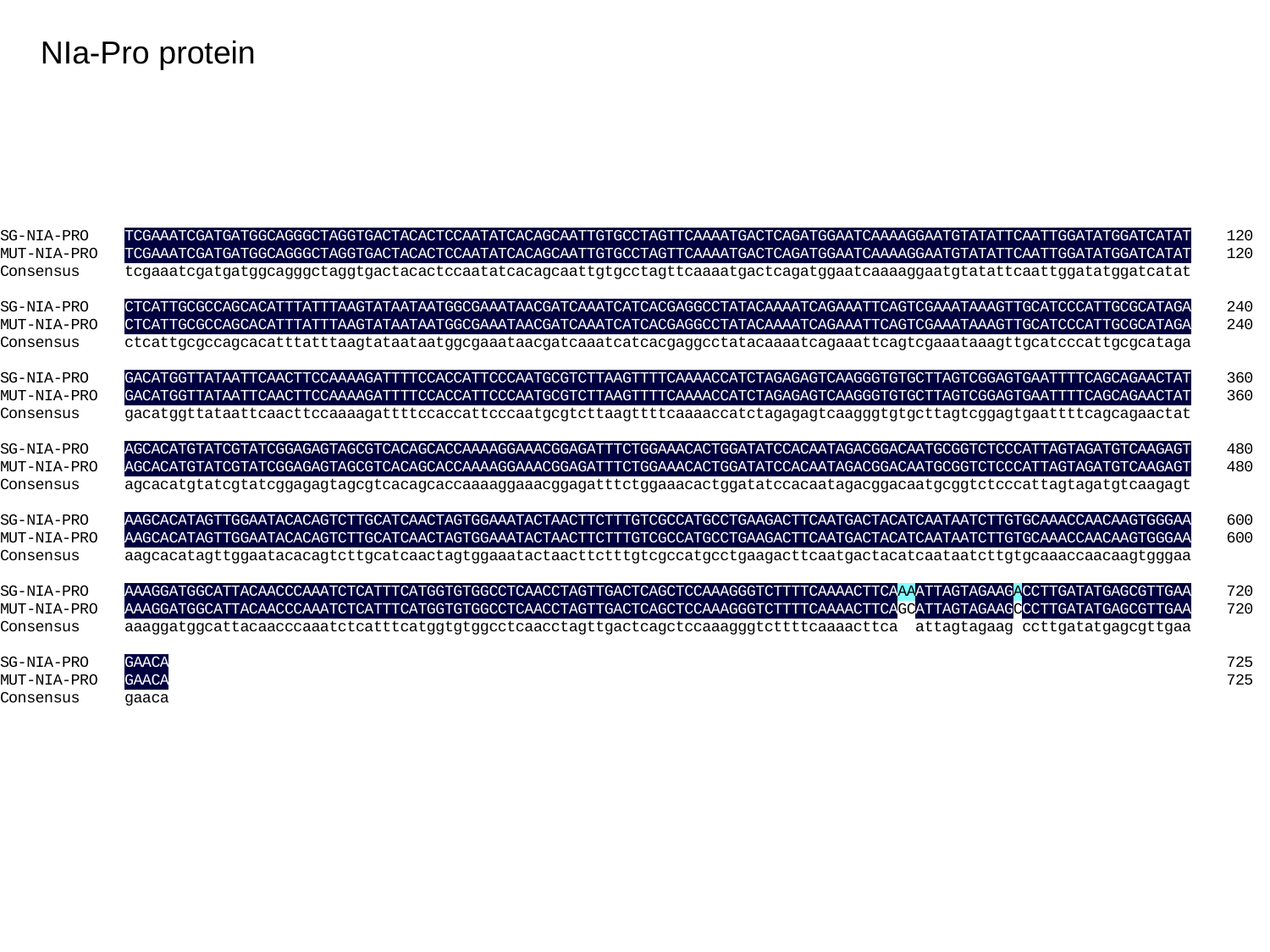

NIa-Pro protein

## Slide 10
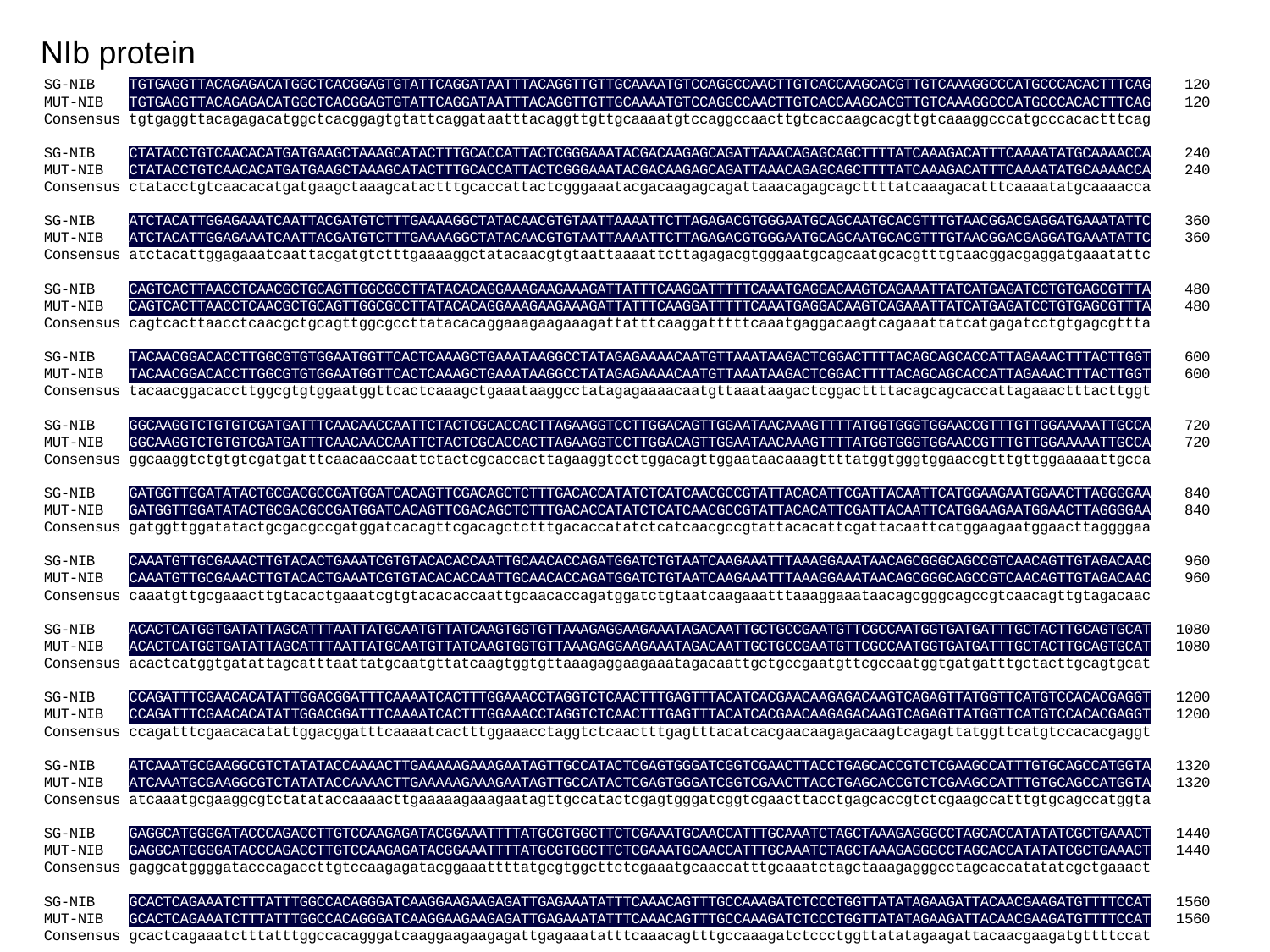

NIb protein

## Slide 11
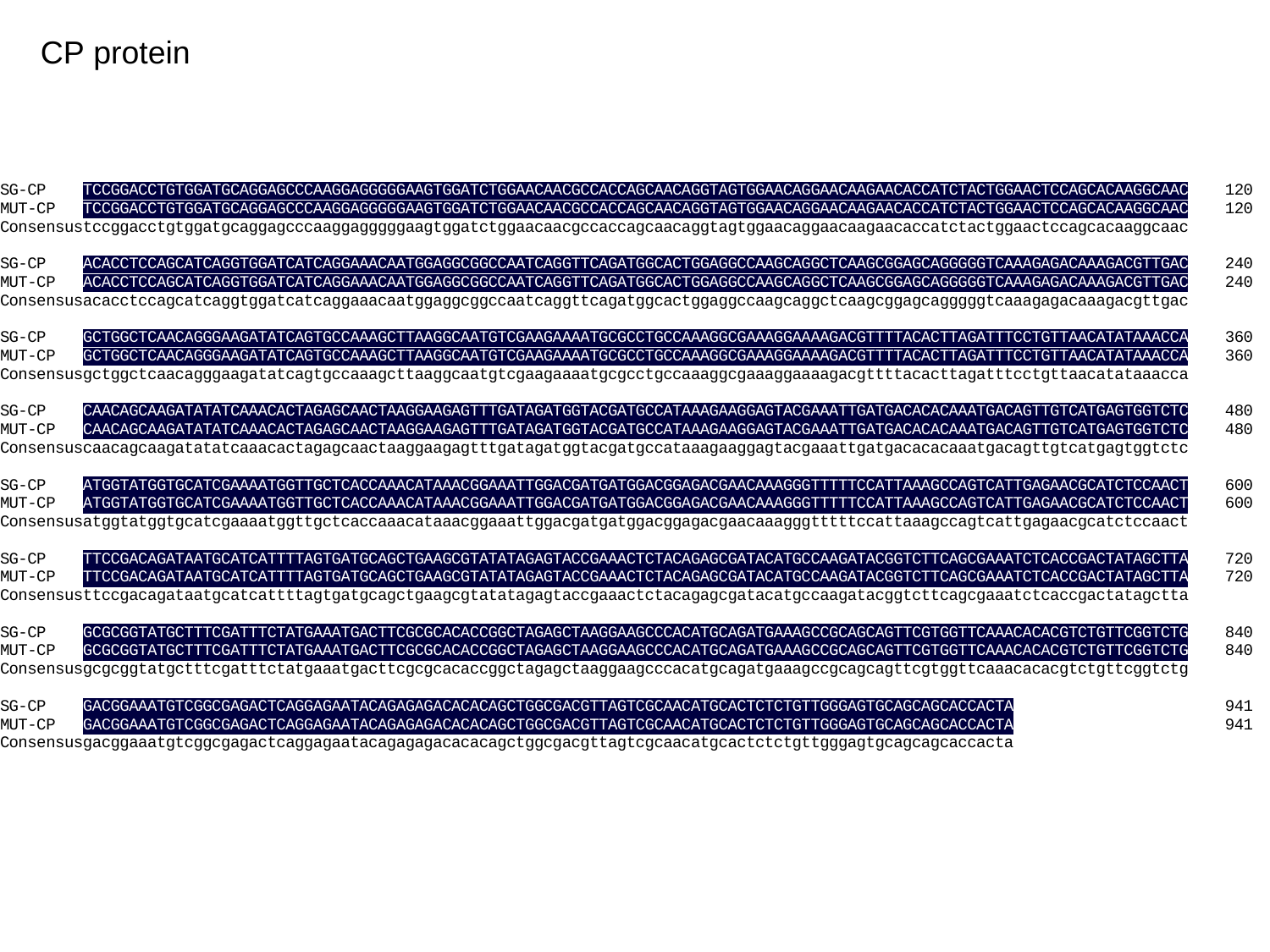

CP protein
