# Supplementary material for: NIa-Pro of sugarcane mosaic virus targets Corn Cysteine Protease 1 (CCP1) to undermine salicylic acid-mediated defense in maize
Source: PLoS Pathog. 2024 Mar 14;20(3):e1012086. doi: 10.1371/journal.ppat.1012086 (PMC10965072; doi:10.1371/journal.ppat.1012086)
Supplement: Attachment — Submitted filename: Response to Reviewers-0115.docx [file ppat.1012086.s026.docx]

**Response to Reviewers' comments**

**Dear Editors and Reviewers,**

Thank you sincerely for dedicating your time to reviewing our manuscript. We are grateful for the opportunity to submit our revised paper and appreciate the valuable feedback provided. Your insightful comments and suggestions have been instrumental in improving our work, and we are pleased that you find this study to be of interest.

With substantial revision, we have thoroughly addressed your comments in the revised manuscript. We extend our gratitude to the reviewers for their diligence and constructive suggestions during the initial review process. Their efforts have significantly contributed to the enhancement of our manuscript.

Please find our **point-by-point responses** to the reviewers' comments in below, with our responses highlighted **in blue**. Moreover, all the revised texts are in blue, and the revised figures are blue-boxed in the submitted files.

We sincerely appreciate the opportunity to submit the revised version of our manuscript. Your continued support and feedback are invaluable to us.

Thank you for your time and consideration.

Best regards,

Tao Zhou

(On behalf of all co-authors)

**-------------------------**

**Point-by-point responses**

**Reviewer #1:**

**Major Issues**

1. One of the main concerns is that none of the gels show the migration of molecular mass markers. Original uncropped gels are not included. Thus, it is very difficult to independently assess that the labeled bands do indeed correspond to specific proteins (also because the calculated molecular mass of the different proteins is not provided). This is particularly a concern in some figures in which unlabeled additional bands are noted. For example, what is the unlabeled predominant lower band in the His Abs blot (pull-down) in Fig 4E? All gels from ALL figures, need to be labeled with the migration of molecular mass markers so that the reader can assess the migration of all bands in relation to the calculated molecular mass of the expected proteins. I also noted a probable labeling error in Fig S5, where the first lane should be positive for GUS 3Myc and NIa-Pro 3Flag but not for preCCP1 3 Myc (otherwise the relative migration of the bands do not make sense).

**Response**:

Thanks for your valuable suggestions. We have already incorporated the protein marker into all the protein gel images in this study as you advised. All modified images have been highlighted with blue boxes for your better viewing of our modifications.

In the process of conducting the GST-pull down experiment, we employed the His-specific antibody to detect TF-mCCP1 (protein size approximately 80 kD). However, we observed consistent detection of bands with molecular weight matching that of GST-NIa-Pro (protein size approximately 50 kD) in all combinations expressing GST-NIa-Pro (including the single expression sample group). This indicates that the His antibody exhibits non-specific binding to the GST-tagged protein. We have reannotated the nonspecific bands in the revised Fig 4E (also shown here).

**Fig 4. SCMV NIa-Pro interacts with the mature protease domain of CCP1 (mCCP1).** A) A schematic diagram showing the domain structure of CCP1. CCP1 contains a signal peptide (SP), an autoinhibitory prodomain (pro-domain) and a mature protease domain. Position of the catalytic triad (Cys, His and Asn) in the mCCP1 is indicated. Different forms of CCP1 are named as preCCP1, iCCP1, and mCCP1, respectively. B) Pair-wise direct Y2H assay was conducted to examine the interaction between mCCP1 and the individual SCMV-encoded proteins. Yeast cells grown on the QDO selective medium are the cells with a positive protein–protein interaction. Yeast cells grown on the DDO medium are the transformed cells. Yeast cells transformed with the empty vector (BD) are used as a negative control. BD, Gal4 Binding Domain; AD, Gal4 activation domain. The AD-T+BD-53 serves as the positive interaction control. C) Co-immunoprecipitation (Co-IP) assay was conducted to verify the interaction between mCCP1 and NIa-Pro in *N. benthamiana* leaves. Mutant mCCP1_C161A,H303A,N330A_ has three alanine substitutions at its catalytic triad. 3Flag-NIa-Pro was co-expressed with mCCP1-3Myc or mCCP1_C161A,H303A,N330A_-3Myc in *N. benthamiana* leaves through agro-infiltration. Leaf tissues were harvested at 48 hours post agroinfiltration (hpai) for Co-IP analysis. Samples of plants co-expressing 3Flag-NIa-Pro and mRFP-3Myc were used as a negative control. Co-IP assay was performed using an anti-Flag affinity agarose gel. Protein samples (Input) and the immunoprecipitated protein samples (Flag-IP) were analyzed through immunoblotting assays using an anti-Flag or an anti-c-Myc antibody. D) Luciferase (Luc) complementation imaging (LCI) analysis of the interaction between NIa-Pro and mCCP1 in *N. benthamiana* leaves. The indicated plasmid pairs were transiently co-expressed in *N. benthamiana*. NLuc co-expressed with CLuc or CLuc-NIa -Pro, and mCCP1-NLuc co-expressed with CLuc, were used as negative controls. The luciferase activity was measured using a luminometer at 48 hpai. E) *In vitro* pull-down assay using a GST-tagged NIa-Pro to immunoprecipitate TF-tagged mCCP1 or mCCP1_C161A,H303A,N330A_. GST-NIa-Pro was used as a bait in this assay and TF-mCCP1 or TF-mCCP1_C161A,H303A,N330A_ was used as a prey. The input and the pull-down protein samples were analyzed through immunoblotting assays using an anti-GST or an anti-His antibody. *, the protein bands corresponding to the expected TF-mCCP1. Purified GST was used as a negative control.

I sincerely apologize for the error in the labeling of Fig S5. The corrected labels have been updated in the revised Fig S7 (also shown here).

**S7 Fig.** **Co-immunoprecipitation (Co-IP) assay was conducted to verify the interaction between preCCP1 and NIa-Pro in *N. benthamiana* leaves.** 3Flag-NIa-Pro was co-expressed with preCCP1-3Myc or GUS-3Myc in *N. benthamiana* leaves through agro-infiltration. Leaf tissues were harvested at 48 hours post agroinfiltration (hpai) for Co-IP analysis. Samples from plants co-expressing 3Flag-NIa-Pro and GUS-3Myc were used as a negative control. Co-IP assay was performed using an anti-Flag affinity agarose gel. Protein samples (Input) and the immunoprecipitated protein samples (Flag-IP) were analyzed through immunoblotting assays using an anti-Flag or an anti-c-Myc antibody.

2. For all quantification analyses (levels of various proteins, activity of CCP1), a gel with the levels of the control actin protein is presented. Were the actin levels taken into account when calculating relative concentrations of CCP1, viral CP as well as the activity of CCP1? This is not clearly explained in the methods. Please provide more details.

**Response**：Thanks very much for your this concern. Indeed, we have taken into account the levels of actin when calculating relative concentrations of CCP1, viral CP as well as the activity of CCP1. For quantification analysis, we firstly normalized the bands of actin, followed by individual calculations for CCP1, SCMV CP, and enzyme activity bands, and then conducted statistical analysis. This information has been added in the revised figure caption (Figs 1 and 2) and Materials and methods section (lines 658-660).

3. The impact of the double mutation (K230A, D234A) is not completely clear (lines 420-427). Although the mutations prevent interaction of NIa-Pro with CCP1 (Fig. 6D) and the inhibition of CCP1 activity (Fig 6E), they do not impact the level of infection of the infectious clone in N benthamiana (Fig 7A-B), but do impact infection in maize after re-inoculation of the N benthamiana infected extracts (Fig 7D-E). Please explain better these discrepancies and rephrase the conclusions more carefully. In fact, I am wondering if the virus sequence was confirmed after passage in N benthamiana and before reinoculation of maize, to confirm that there were no additional mutations that may have been introduced upon passage in the first host.

**Response**：Thanks for your critical comments. On *N. benthamiana*, SCMV only shows local infection. To dissect why the mutations of NIa-Pro do not impact the multiplication of SCMV in *N. benthamiana*, we searched for the homologous gene *NbCYP2* of *ZmCCP1* in *N. benthamiana*. Our yeast two-hybrid analysis revealed no interaction between SCMV NIa-Pro and mature peptide of NbCYP2 or AtRD19a (homologous gene of *CCP1* in *A. thaliana)* (as shown below). Thus, we speculate that the interaction between NIa-Pro and CCP1 may not necessarily lead to successful SCMV infection in dicotyledonous plants. This can explain the observations from our inoculation experiment that the infection levels of SCMV-GFP and the double mutant virus SCMV-NIa-Pro_K230A, D234A_-GFP in *N. benthamiana* leaves are comparable. However, upon transfer into maize, noteworthy differences become apparent between the double mutant virus SCMV-NIa-Pro_K230A, D234A_-GFP and SCMV-GFP.

**Y2H assay shows that SCMV NIa-Pro does not interact with the mature protease domain of CCP1 analogue NbCYP2 in *N. benthamiana* or AtRD19A in *A. thaliana***. The AD-T+BD-53 serves as the positive control.

Moreover, we conducted three additional inoculation attempts, which ultimately revealed a significantly lower incidence of disease caused by the double mutant virus compared to SCMV-GFP in maize. The detailed information is shown in the revised manuscript (lines 391-432) and revised Figs 7 and 8 (also shown here).

**Fig 7. Truncated NIa-Pro or key amino acid mutants NIa-Pro_K230A, D234A_ both inhibited SCMV infection in maize plants.** A) GFP analysis of the *N. benthamiana* leaves agro-infiltrated with the wild type virus SCMV-GFP, the inhibitor motif of NIa-Pro-defective mutant SCMV-NIa-Pro_IMD_-GFP and substitutions mutant SCMV-NIa-Pro_K230A, D234A_-GFP at 5 dpai. Under UV light, strong GFP fluorescence was evident in pSCMV-GFP and pSCMV-NIa-Pro_K230A, D234A_-GFP infected areas. No GFP fluorescence was observed on pSCMV-NIa-Pro_IMD_-GFP infected area. B) RT-qPCR analysis of the relative accumulation levels of SCMV RNA in the SCMV agro-infiltrated leaves at 5 dpi. Error bars represent the means ± SE. Statistical differences (**, *P* < 0.01) were evaluated by Student’s *t* test analysis. Three independent experiments are conducted. C) Statistical analysis of the incidence of three different viruses on maize plants. D) Symptoms of maize plants infected with SCMV-GFP, SCMV-NIa-Pro_IMD_-GFP or SCMV-NIa-Pro_K230A, D234A_-GFP at 8 dpi. Bar = 5 cm.

**Fig 8. The double mutant virus SCMV-NIa-Pro_K230A, D234A_-GFP fails to induce CCP1 gene expression and activates PLCP activity.** A) RT-qPCR analysis of the relative accumulation levels of SCMV RNA in the SCMV-GFP- or SCMV-NIa-Pro_K230A, D234A_-GFP- infected first systemically leaves at 8 dpi. B) Immunoblotting analysis of SCMV CP accumulation in the first systemically infected maize leaves. C) RT-qPCR analysis of the relative accumulation levels of *CCP1* in the mock, SCMV-GFP- or SCMV-NIa-Pro_K230A, D234A_-GFP- infected first systemically leaves. D) Immunoblotting analysis of CCP1 accumulation and activity-based protein profiling (ABPP) analysis of the protease activities of maize PLCPs in the first systemically infected leaves at 8 dpi. E) The contents of SA in the mock, SCMV-GFP- or SCMV-NIa-Pro_K230A, D234A_-GFP- infected first systemically leaves. F) RT-qPCR analysis of the relative accumulation levels of *PR1* and *PR5* in the mock, SCMV-GFP- or SCMV-NIa-Pro_K230A, D234A_-GFP- infected first systemically leaves. Error bars represent the means ± SE. Statistical differences (*, *P* < 0.05; **, *P* < 0.01; ***, *P* < 0.001; ****, *P* < 0.0001) were evaluated by two-tailed Student’s *t* test analysis. Three independent experiments are conducted.

Finally, we conducted a sequencing analysis of genome of the mutant virus SCMV-NIa-Pro^K230A, D234A^-GFP extracted from *N. benthamiana* leaves. The result confirmed the expected two amino acid mutations in NIa-Pro. In addition, there have also been several nucleotide mutations in the *P1*, *HC-Pro*, and *VPg* coding regions. However, upon analysis, these mutations in the *P1*, *HC-Pro*, and *VPg* coding regions were all nonsense mutations. Therefore, it can be confirmed that no other missense mutations were present in the mutant virus before its passage through *N. benthamiana* and subsequent inoculation in maize. Please refer to the supplement information 2 (The sequencing analysis of the SCMV mutant prior to the inoculation of maize) to see the details.

**Minor Issues**

 1. Lines 137-138, based on Fig. 1E, the surge of PLCP activity was only significantly at 7 dpi and not at 12 dpi. Also, line 140, since the impact of SCMV infection is dependent on the timing of infection, rather than stating that SCMV infection “amplifies” maize PLCPs, it is more accurate to state that it “alters” the regulation of maize PLCPs.

**Response**： Thanks for your suggestions. We made revisions on lines 163 and 165, following the suggestions.

2. Line 84-85: This sentence needs references.

**Response**：Thanks. As you suggested, we have reinstated the citation within the sentence on lines 109-111.

3. Lines 196-199. It may be useful to explain what forms of CCP1 are normally found in plants. I am assuming that the mature protease is normally released from the precursor by autocatalytic cleavage. Has this been demonstrated (either for this protease or for related proteases)? If so, please provide references. What are the biological functions of the precursor and mature forms of CCP1?

**Response**：Thanks for your valuable suggestion. As depicted in Fig 4A, there are typically three forms of CCP1 present in plants: the precursor of CCP1 (preCCP1), the CCP1 with the signal peptide removed but containing the enzyme-inhibitory domain (iCCP1), and the mature form of CCP1 (mCCP1). While there is no direct research evidence on the activation of CCP1, taking the example of PLCP RD21 in *A. thaliana*, it includes not only the N-terminal signal peptide and enzyme auto-inhibitory domain but also the C-terminal granulin domain. When expressed in *N. benthamiana*, four distinct forms of RD21can be detected, however, it is only activated upon removal of the signal peptide, the enzymatic inhibition domain, and the C-terminal granulin domain [1, 2]. We speculate that CCP1 requires two steps to remove the signal peptide and auto-inhibitory domain, ultimately activating to its mature protease form. Therefore, both the precursor and the form with the signal peptide removed of CCP1 are in an activity-inhibited state, and only the mature form of CCP1 exhibits protease activity. We have provided additional explanation in the revised main text (on the lines 240-244).

Related references:

1. Shindo T, Misas-Villamil JC, Horger AC, Song J, van der Hoorn RAL. A role in immunity for Arabidopsis cysteine protease RD21, the ortholog of the tomato immune protease C14. PLoS One. 2012;7(1). doi: ARTN e29317 10.1371/journal.pone.0029317. PubMed PMID: WOS:000301188800012.
2. Shindo T, Kaschani F, Yang F, Kovacs J, Tian F, Kourelis J, et al. Screen of non-annotated small secreted proteins of Pseudomonas syringae reveals a virulence factor that inhibits tomato immune proteases. PLoS Pathog. 2016;12(9). doi: ARTN e1005874 10.1371/journal.ppat.1005874. PubMed PMID: WOS:000385621900036.

4. Lines 218-219. It is also possible that the triple mutation of the catalytic triad changed the overall conformation of the protein and altered the presentation of another interacting domain.

**Response**：Thanks for your critical comment. In order to anticipate the impact of triple mutation of the catalytic triad of CCP1, we simulated and predicted the structure separately (revised Fig S12, also shown here). The results indicate that the mutations have no effect on the protein conformation. We also added additional explanation in the revised main text (on the lines 264-267).

**S12 Fig.** **Three-dimensional model of mCCP1 (A) and mutant mCCP1_C161A,H303A,N330A_ (B), in cartoon style.**

5. Lines 270-272. This part of the text is not very clear, what is meant by “expressed fewer heterologous proteins”? What are those heterologous proteins?

**Response**：Many thanks. To determine the suppression role of NIa-Pro on the protease activity of PLCPs, we employed a FoMV vector to express NIa-Pro, with expressing GFP as negative control. The phrase "expression of a lesser amount of exogenous protein" is indicative of the utilization of the FoMV system to overexpress NIa-Pro or control protein GFP. We rewrote the sentence for concise and clear. That is “**The results showed that, though the accumulation level of 3Flag-NIa-Pro was significantly lower than that of 3Flag-GFP, PLCP activity was markedly reduced in NIa-Pro-expressed (FoMV-3Flag-NIa-Pro) plants compared to the control (FoMV-3Flag-GFP) plants (Fig 5C and 5D)**.” (on the lines 328-332).

6. Lines 284: the C-terminal domain is introduced as an inhibitor motif. Is it related to other known inhibitor motifs, or is this statement only based on experiments that are described later in the manuscript? If there was no previous known inhibitor activity of this C-terminal domain, it may be better to state that the C-terminal domain has an unknown function but could potentially act in the CCP1 inhibition since it is surface-localized. Is the C-terminal inhibitor domain also present in other 3C-like proteases from plant or animal picorna-like viruses or is it specific to potyviruses?

**Response**：

Thanks very much for this concern. We have predicted the three-dimensional conformation of the NIa-Pro protein, and found that the C-terminal sequence is located on the surface of the NIa-Pro protease pocket, and its presence significantly occludes the protease pocket. Therefore, it is hypothesized to be an enzyme activity inhibitor structure.

Following your advice, we have discovered that several literature reports suggest that the C-terminus of NIa-Pro encoded by TEV (tobacco etch virus) binds to the active site of NIa-Pro [3]. The full-length form of NIa-Pro encoded by TVMV (tobacco vein mottling virus) exhibits significantly lower activity than its C-terminus-deleted form [4], suggesting that the small peptide at the C-terminus of NIa-Pro might potentially exert inhibitory effects on the activity of NIa-Pro. We also added additional explanation in the revised main text (on the lines 340-347). Thus, we speculate that the C-terminus of SCMV-encoded NIa-Pro plays a role in interacting with CCP1 and inhibiting the protease activity.

In addition, upon literature research, we found that although the NIa-Pro of potyviruses shares structural similarity with the 3C-Pro protein of picorna-like viruses, the identity on amino acid sequence is low (38.41%). The 3C-Pro of picorna-like viruses also contains a C-terminus, but it has not been reported whether it possesses the function of inhibiting the activity of 3C-Pro [5, 6].

Related references:

3.	Nunn CM, Jeeves M, Cliff MJ, Urquhart GT, George RR, Chao LH, Tscuchia Y, Djordjevic S. Crystal structure of tobacco etch virus protease shows the protein C terminus bound within the active site. J Mol Biol. 2005;350(1). doi: 10.1016/j.jmb.2005.04.013.

4.	Sun P, Austin BP, Tözsér J, Waugh DS. Structural determinants of tobacco vein mottling virus protease substrate specificity. Protein Sci. 2010;19(11). doi: 10.1002/pro.506.

5.	Ekanayaka P, Weerawardhana A, Chathuranga K, Park JH, Lee JS. Foot-and-mouth disease virus 3Cpro cleaves BP180 to induce blister formation. Viruses. 2022;14(9). doi: 10.3390/v14092060.

6.	Wan L, Wang X, Wang T, Yuan X, Liu W, Huang Y, Deng C, Cao S. Comparison of target pocket similarity and progress into research on inhibitors of picornavirus 3C proteases. Chem Biodivers. 2023;20(3). doi: 10.1002/cbdv.202201100.

7. Lines 295 and Fig S12 (also lines 420-423). These sections of the text are not clear. In Fig S12, five amino acids are highlighted as being completely conserved in all NIa-Pros. The reader assumes that the five amino acids mutated correspond to the strictly conserved amino acids, but this is not the case. Residues N206, K230 and D234, are not strictly conserved in all potyvirus NIa-Pro, but were chosen for the mutagenesis analysis and the last two did show an impact on the ability of NIa-Pro to interact with CCP1. All mutated residues should be labeled in Fig S12 and a better explanation should be provided as to why these amino acids were chosen for the mutagenesis analysis.

**Response**：Thanks for your comment. We have modified Fig S12 (renamed as S19 in the revised version) and reannotated the five amino acid residues undergoing mutation. While W199 and W203 are strictly conserved in the aligned *Potyvirus* species, residues N206, K230, and D234, although not strictly conserved, are conserved in the majority of the viruses. Additionally, through the docking analysis of the protein-protein interaction between NIa-Pro and CCP1, we infer from the structure that residues W199, W203, N206, K230, and D234 are more likely to play crucial roles in their interaction, hence the selection of these sites for mutation. We added this explanation in figure legend of Fig S19 and the revised main text (on lines 356-358).

8. Lines 301-303. I could not figure out which lane corresponds to the in vitro synthesized 48 aa inhibitor peptide in Fig. 6E. Do the authors mean that the inhibitor domain was fused with the GST (construct GST-imNIa-Pro?)

**Response**：I sincerely apologize for the mistake, as the synthetic peptide consisting of 48-amino acid inhibitor did not exhibit significant activity. Hence, we used purified fusion protein of GST-imNIa-Pro for protease activity experiments. We have made the appropriate modifications in the main text (on line 367), and labeled Fig 6E with the migration of molecular mass markers.

9. Lines 316-318, change “areas expressing SCMV” for “areas infected with SCMV” (two instances)

Reply：Thank you for your suggestion. We have made modifications in the revised main text (on lines 381, 383, 384, 386, and 387).

10. Line 408, it is stated that NIa-Pro binds but cannot cleave CCP1. The sentence is not clear. Is it meant that CCP1 does not cleave NIa-Pro to release it from the catalytic site of CCP1, or is it meant that NIa-Pro does not cleave CCP1.

**Response**：Thanks for this concern. We have observed that the protein stability of CCP1 is hardly affected when it is co-expressed with NIa-Pro (revised Fig S17). Moreover, no significant degradation of recombinant TF-mCCP1 was observed when it was co-incubated with the recombinant GST-NIa-Pro (Fig 4E). Hence, we conclude that NIa-Pro is capable of binding to CCP1 without cleaving it. We revised this sentence as “**Our data support that NIa-Pro could bind to CCP1, but cannot cleave CCP1, thus obstructing the CCP1 catalytic triad**.” in line 518-519.

11. Lines 433-445. Please refer to Fig 8 and explain in the text that this is a proposed model.

**Response**：Thanks for your valuable suggestion. We refer to Fig 8 (revised Fig 9) and provided an explanation in the text (on the lines 436-441).

12. Fig. 1 legend, 3rd line. Replace “phosphorate” by “phosphate”

**Response**：Thanks for your suggestions, we have already made this correction in the revised Fig 1 legend.

13. Fig. 3 legend. Please explain what the 4 stars represent in terms of P values.
**Response**：Sorry for this missing. In statistical analysis, a *P*-value less than 0.0001 is denoted with four asterisks. We have made the necessary addition in the revised Fig 3 legend.

14. Fig. 4 legend. Please explain what the AD-T+BD-53 constructs are. Positive control?

**Response**：I apologize for not clarifying in the caption. Indeed, the AD-T+BD-53 combination serves as the positive interaction control in the yeast two-hybrid system, and we have made the necessary addition in the figure legend.

**Reviewer #2:**

**Major Issues**

1. Authors state that CCP1 plays a role in modulating SA signaling via knocking down this gene. Moreover, NIa-Pro suppresses CCP1’s protease activity to dampen plant defense. Whether CCP1’s protease activity is important for SA signaling and plant defense against SCMV infection has not been investigated here, although similar mechanism has been reported in maize previously. These two parts seems unrelated currently. Authors should use this mutant mCCP1C161A, H303A, N330A to further validate this question. Also, can CCP1 directly target SCMV proteins for cleavage to suppress SCMV infection?

**Response**：Thanks for your valuable suggestion. We conducted transient overexpression experiments in maize protoplasts, and the results demonstrated that the overexpression of *CCP1* inhibits the accumulation of SCMV RNA (as shown in revised Fig S5). However, overexpression of its enzymatic activity-deficient mutant *CCP1_C161A, H303A, N330A_* failed to suppress SCMV proliferation (revised Fig S5, lines 188-201 of revised main text). These findings indicate the defensive role of CCP1 proteinase activity against SCMV infection in maize. We have incorporated this section into the main text, please referred to lines 188-201 in the revised manuscript. We have also examined the expression of the SA marker genes *PR1* and *PR5* in protoplasts overexpressing CCP1, and found a both significant increase (as shown in revised Fig S5, also shown here), which suggests up-regulated SA signaling.

Transient overexpression experiments in maize protoplasts showed that activity of CCP1 is crucial for inhibiting the accumulation of SCMV RNA. To further explore the changes of SA signaling pathway and corresponding resistance, it is necessary to construct transgenic maize overexpressing CCP1 or the mutant CCP1_C161A, H303A, N330A_. As it will take two more years to obtain transgenic maize for overexpression, we claim to not to do this.

In addition, our experimental findings indicate that the full-length CCP1 indeed impacts the stability of NIa-Pro (as shown in revised Fig S18, lines 521-524); however, there is a lack of direct evidence to substantiate the cleavage of NIa-Pro or any other viral protein by CCP1.

**S5 Fig. Over-expression of CCP1 in maize protoplasts suppresses SCMV RNA replication.** Protoplasts were separately co-transfected with a mixture of pGD-CCP1-mRFP and SCMV RNA, a mixture of pGD-mRFP and SCMV RNA, or a mixture of pGD-CCP1_C161A,H303A,N330A_-mRFP and SCMV RNA. The expression level of *CCP1* (A), the relative accumulation of SCMV RNA (B) and the expression level of SA marker genes *ZmPR1* (C) and *ZmPR5* (D) were determined at 18 h post transfection. Three independent experiments were conducted with at least three biological replicates per treatment. Error bars are the means ± SE. **, *P* < 0.01; ***, *P* < 0.001; ****, *P* < 0.0001; determined by the unpaired t-test.

2. NIa-Pro interacts with CCP1, but how this interaction affects CCP1’s function, especially its protease activity, is still largely unknown. Why NIa-Pro? Subcellular localization or protein accumulation change? Actually, we can see CCP1 protein accumulation is indeed decreased under SCMV infection at early infection stages shown in Fig.1B.

**Response**：Thanks for your this concern. In this study, our data suggested that NIa-Pro could interact with CCP1 to inhibit the protease activity. Utilizing the yeast two-hybrid system for potential interaction screen identified that solely NIa-Pro interacts with CCP1 (Fig 4B). Moreover, LCI, Co-IP and GST pull-down assays confirmed the interaction of NIa-Pro with CCP1. Their colocalization observed within the lytic vacuole suggested the potential biological function for their interaction (as shown in revised Fig S16). Upon co-expression in *N. benthamiana* plants, it has been observed that NIa-Pro does not significantly affect the stability of CCP1 in either its precursor or mature peptide forms (as shown in revised Fig S17, lines 291-295 of revised main text). However, our protease profile assays have shown that NIa-Pro significantly inhibits the enzymatic activity of CCP1 (Fig 5). Taken together, these findings suggested that NIa-Pro could interact with CCP1 to inhibit the protease activity.

Regarding the question of why the CCP1 protein levels are downregulated during the early stages of viral infection, this can be elucidated through the activation of CCP1. During the early stages of viral infection, the CCP1 protein band detected corresponds to the precursor of CCP1 (Fig 1B). A decrease in the precursor protein band indicates an increase in the mature enzymatic form, thereby enhancing resistance. In the later stages of SCMV infection, the binding of SCMV NIa-Pro inhibits CCP1 enzyme activity, thus facilitating a long-term infection on maize.

3. Truncated NIa-Pro and its mutants can inhibit SCMV infection, which may not be caused by its deficit in suppressing CCP1 activity. As NIa-Pro is the main protease for potyviral polyprotein processing and also has other important functions, authors should test whether these NIa-Pro mutants used in Fig.7 still harbour normal protease activity et al. Also, SA level, PLCP enzyme activity and CCP1 expression should be investigated in plants infected with SCMV mutants compared with WT SCMV.

**Response**：Thanks for your comments. We conducted three additional inoculation attempts, which ultimately revealed a significantly lower incidence of disease caused by the double mutant virus SCMV-NIa-Pro_K230A, D234A_-GFP compared to SCMV-GFP in maize. However, the SCMV-NIa-Pro_IMD_-GFP did not show any symptoms even at 15 dpi (as shown in revised Fig 7, also shown here). We have revised the section according to the updated inoculation assay findings on lines 391-400.

**Fig 7. Truncated NIa-Pro or key amino acids mutant NIa-Pro_K230A, D234A_ inhibited SCMV infection in maize plants.** A) GFP analysis of the *N. benthamiana* leaves agro-infiltrated with the wild type virus SCMV-GFP, the inhibitor motif of NIa-Pro-defective mutant SCMV-NIa-Pro_IMD_-GFP, and substitutions mutant SCMV-NIa-Pro_K230A, D234A_-GFP at 5 dpai. Under UV light, strong GFP fluorescence was evident in pSCMV-GFP and pSCMV-NIa-Pro_K230A,D234A_-GFP infected areas. No GFP fluorescence was observed on pSCMV-NIa-Pro_IMD_-GFP infected areas. B) RT-qPCR analysis of the relative accumulation levels of SCMV RNA in the SCMV agro-infiltrated leaves at 5 dpi. Error bars represent the means ± SE. Statistical differences (**, *P* < 0.01) were evaluated by Student’s *t* test analysis. Three independent experiments are conducted. C) Statistical analysis of the incidence of symptoms appearance on maize plants. D) Symptoms of maize plants infected with SCMV-GFP, and its NIa-Pro mutants SCMV-NIa-Pro_IMD_-GFP or SCMV-NIa-Pro_K230A, D234A_-GFP at 8 dpi. Bar = 5 cm.

To investigate whether the proteolytic activity of NIa-Pro SCMV mutants is compromised, we employed antibodies specific to various SCMV-encoded proteins to detect differing viral proteins within the mutants. Experimental findings indicate the presence of intact viral proteins in the mutant SCMV-NIa-Pro_K230A, D234A_-GFP-infected maize plants, suggesting that the NIa-Pro proteinase activity of SCMV-NIa-Pro_K230A, D234A_-GFP is unimpaired (Please refer to the revised Fig S21, also shown here). Due to the absence of apparent infection in *N. benthamiana* local leaves and lack of successful infection in maize of the mutant virus SCMV-NIa-Pro_IMD_-GFP, we hypothesize that the absence of the C-terminal inhibitory peptide of NIa-Pro affects the function of NIa-Pro.

**S20 Fig. The expression of several SCMV-encoded proteins in SCMV-GFP- or its double mutant variants SCMV-NIa-Pro_K230A, D234A_-GFP-infected maize leaves.** At 7 dpi, leaf samples were collected for immunoblot analysis using specific antibodies to detect the expression of SCMV HC-Pro (A), CI (B), NIa-Pro (C), and CP (D).

In the revised manuscript, we have added the section including analysis of the levels of SA, PLCP enzyme activity, and CCP1 expression in maize plants infected with the double mutant virus SCMV-NIa-Pro^K230A, D234A^-GFP compared to the mock group and wild-type virus SCMV-GFP. Please find the detailed information on lines 402-432 of revised manuscript and revised Fig 8 (also shown here).

**Fig 8. The double mutant virus SCMV-NIa-Pro_K230A, D234A_-GFP fails to induce CCP1 gene expression and activates PLCP activity.** A) RT-qPCR analysis of the relative accumulation levels of SCMV RNA in the SCMV-GFP- or SCMV-NIa-Pro_K230A, D234A_-GFP- infected first systemically leaves at 8 dpi. B) Immunoblotting analysis of SCMV CP accumulation in the first systemically infected maize leaves. C) RT-qPCR analysis of the relative accumulation levels of *CCP1* in the mock, SCMV-GFP- or SCMV-NIa-Pro_K230A, D234A_-GFP-infected first systemically leaves. D) Immunoblotting analysis of CCP1 accumulation and activity-based protein profiling (ABPP) analysis of the protease activities of maize PLCPs in the first systemically infected leaves at 8 dpi. E) The contents of SA in the mock, SCMV-GFP- or SCMV-NIa-Pro_K230A, D234A_-GFP-infected first systemically leaves. F) RT-qPCR analysis of the relative accumulation levels of *PR1* and *PR5* in the mock, SCMV-GFP- or SCMV-NIa-Pro_K230A, D234A_-GFP-infected first systemically leaves. Error bars represent the means ± SE. Statistical differences (*, *P* < 0.05; **, *P* < 0.01; ***, *P* < 0.001; ****, *P* < 0.0001) were evaluated by two-tailed Student’s *t* test analysis. Three independent experiments are conducted.

4. The effect of NIa-pro and these mutants on plant defense needs to be investigated. Also, if NIa-Pro indeed target CCP1 to dampen SA-mediated host defense, genetic evidences are lacking.

**Response**：Thanks for your constructive comments. In our experiment, we expressed NIa-Pro using a FoMV vector, in comparison to the control group which expressed GFP. The level of active PLCPs was significantly decreased in the group expressing NIa-Pro (Fig 5C). Regrettably, our subsequent challenge inoculation of SCMV on the FoMV-infected maize plants failed. However, we have observed a significant reduction in virulence of the mutant virus SCMV-NIa-Pro_K230A, D234A_-GFP with mutated key residues responsible for NIa-Pro–CCP1 interaction. Please find the detailed information on lines 391-432 of revised manuscript and revised Figs 7 and 8 (also shown in our response to the comment 3 above). In addition, the expression levels of CCP1 and the SA signaling pathway are not induced in maize plants infected with the mutant virus SCMV-NIa-Pro_K230A, D234A_-GFP.

**Minor Issues**

1. In fig.1A and B, relative RNA and protein accumulation levels of CCP1 are not consistent between two treatments, and even contrary. Also, the PLCP activities is not consistent with CCP1 accumulation level. For example, under SCMV infection at 7 dpi, CCP1 is significantly downregulated (1B), however, PLCP activities are upregulated (1D), compared with mock. The quantified results in Fig.1E are also not consistent with 1D (10 and 12 dpi). CCP1 antibody and gene primer may be not specific, how about CCP2? Authors should provide immunoblot results against SCMV CP in Fig.1B.

**Response**：Thanks for your critical comment. The antibodies against CCP1 and the CCP1-specific primers used for RT-qPCR have been validated for specificity. Initially, we observed inconsistent and even opposite accumulation patterns of CCP1 RNA and protein levels following SCMV infection, leading us to speculate that viral infection may have a global impact on host regulation at the transcription, translation, and post-translation levels. This explains the differential regulation of transcription and translation levels of CCP1 during viral infection.

Additionally, regarding the inconsistencies in the accumulation levels of activated PLCP compared to CCP1, this can be elucidated through the activation of CCP1. During the early stages of viral infection, the CCP1 protein band detected corresponds to the precursor of CCP1 (Fig 1B). A decrease in the precursor protein band indicates an increase in the mature enzymatic form, thereby enhancing accumulation of active PLCPs. Furthermore, we are aware of the presence of PLCPs family in plants, consisting of nine subfamilies. The use of DCG-04 allows us to detect only the total activated form of PLCPs, without the ability to selectively detect specific activated PLCP. Thus, the bands showing activated PLCPs include not only activated CCP1 but also other subfamilies of PLCP, such as CCP2, RD21, etc. We speculate that there is a complex regulation of *PLCP* genes at different levels during viral infection, but ultimately, the functional manifestation is reflected in the level of activated forms. Since this study focuses on CCP1, we have not conducted a detailed study on the function of CCP2.

Following your suggestion, we have incorporated the immunoblot results for SCMV CP in Fig 1B, depicting the accumulation levels of SCMV CP during 5–14 dpi. Please refer to the revised Fig 1B.

2. In fig.2, Authors use CMV-mediated VIGS system to investigate the effect of silencing CCP1 on the second SCMV infection. Since silencing of CCP1 suppressed plant defense, thus, we can speculate that CMV accumulation has been also increased and then SCMV infection in CCP1-silenced plants. Authors should exclude this possibility.

**Response**：Thanks for your valuable suggestion. Following your suggestion, we assessed the accumulation levels of CMV RNA in the CMV-CCP1+SCMV group compared to the CMV-GUS+SCMV group. Unexpectedly, the results showed that the accumulation levels of CMV RNA were similar in both groups, with no significant differences (as shown in revised Fig S4). It is considered that probably because CMV and SCMV belong to distinct viral families. We are sorry that we could not have more explanation on this result.

3. Authors should also test the effect of CCP1 over-expression on SA signaling and SCMV infection in maize protoplasts or leaves.

**Response**：Thanks for your suggestion. We overexpressed CCP1 in maize protoplasts and observed a significant reduction in the accumulation of SCMV RNA and induction of SA responsive-genes *ZmPR1* and *ZmPR5* (as shown in revised Fig S5, lines 188-201 of revised main text). However, the analysis of SA hormone levels in maize protoplasts was restricted due to limited protoplast quantity. To further explore the changes of SA signaling pathway and corresponding resistance in maize leaves, it is necessary to construct transgenic maize overexpressing CCP1 and the mutant CCP1_C161A, H303A, N330A_. As it will take two more years to obtain transgenic maize for overexpression, we claim to not to do this.

4. In Fig.4B, authors use nuclear Y2H system to investigate CCP1 interaction with each of SCMV proteins. Since most of SCMV proteins are membrane and cytoplasm protein, nuclear Y2H is not appropriate.

**Response**：Thanks for your constructive comments. In the initial stages of our experiment, we employed Y2H system to perform pairwise interaction analysis with SCMV proteins interacting with CCP1. Although the drawback of the Y2H system lies in its ability to validate only nuclear protein interactions based on AD/BD, it lacks high credibility in detecting interactions involving proteins located outside the nucleus, membrane proteins, or proteins that undergo subcellular relocalization after interaction. Nevertheless, upon consulting relevant literature on the interaction between plant PLCPs and pathogen-encoded proteins, we found that most of the protein interactions were initially identified using the Y2H screening approach. Additionally, the interaction between NIa-Pro and CCP1, which we screened, has been further confirmed through other experimental methods including LCI, Co-IP and GST-pull down.

5. Lines 202-203: 3×myc, 3×flag. If use partial gus fragment, authors should indicate.

**Response**：Thanks for the suggestion. We utilized the GUS-3×myc constructed with complete GUS fragments and the NIa-Pro-3×flag combination as our negative control. However, there was a labeling error in the accompanying figure. We have revised the labeling of the revised Fig S7 (also shown here).

**S7 Fig.** **Co-immunoprecipitation (Co-IP) assay was conducted to verify the interaction between preCCP1 and NIa-Pro in *N. benthamiana* leaves.** 3Flag-NIa-Pro was co-expressed with preCCP1-3Myc or GUS-3Myc in *N. benthamiana* leaves through agro-infiltration. Leaf tissues were harvested at 48 hours post agroinfiltration (hpai) for Co-IP analysis. Samples from plants co-expressing 3Flag-NIa-Pro and GUS-3Myc were used as a negative control. Co-IP assay was performed using an anti-Flag affinity agarose gel. Protein samples (Input) and the immunoprecipitated protein samples (Flag-IP) were analyzed through immunoblotting assays using an anti-Flag or an anti-c-Myc antibody.

6. Line 421: about-identity?

**Response**：We have incorporated sequence identity 74.44% into the revised version of manuscript (on line 535, also shown here).

Amino acid sequence alignment of NIa-Pros belonging to the different potyviruses shows about **74.44%** identity and there are some conserved residues in the inhibitory motif.

7. Fig.6A, P3N-PIPO, not pipo.

**Response**：Thanks for your comment. We have consulted the relevant literature and found that "pipo" refers to a short open reading frame (ORF) generated through a +2 frameshift within the P3 gene [7]. This ORF has been named PIPO. While it is expressed as a fusion protein in the form of P3N-PIPO during virus infection, we annotate PIPO as the small ORF in Fig. 6A.

Related references:

7.	Chung BY, Miller WA, Atkins JF, Firth AE. An overlapping essential gene in the *Potyviridae*. Proc Natl Acad Sci U S A. 2008;105(15). doi: 10.1073/pnas.0800468105. PubMed PMID: WOS:000255237200045.

8.In Fig.6, authors should also use other methods including co-ip to confirm the protein-protein interaction results. Only LIC assay data is not solid.

**Response**：Thanks for your suggestion. We also employed yeast two-hybrid assay to investigate the interaction between inhibitory region of NIa-Pro with mutated conservatively positioned amino acids and mCCP1. The results were consistent with those obtained from the LCI assay, indicating that residues 230K and 234D are both vital for the NIa-Pro–CCP1 interaction. Please refer to the revised Fig S20 for the detailed results (also shown here). We have also incorporated this yeast double hybrid findings into the revised manuscript (please refer to lines 360-362).

**S20 Fig. Y2H assay shows that residues** **230K and 234D are both vital for the NIa-Pro**–**CCP1 interaction.** Yeast cells grown on the QDO selective medium are the cells with a positive protein–protein interaction. The AD-T+BD-53 serves as the positive control.

**Reviewer #3:**

**Major Issues**

The cell biology should be supported by western blot experiments showing that the fusions are intact. The Nla-Pro-GFP fusion shows a similar localization as free GFP would have, which is missing as a negative control. Likewise, it is hard to imagine that CCP1 would be in the cytoplasm if it has a signal peptide for secretion. Please provide stronger proof that these proteins co-localise and discuss possible mechanisms.

**Response**：Thanks for your suggestions. We have incorporated GFP as a negative control. The results further demonstrated that NIa-Pro-GFP localizes within the cytoplasm and nucleus, similar to the wild-type GFP (As shown in revised Fig S14, also shown here).

**S13 Fig. The localization pattern of SCMV NIa-Pro.** Confocal images of *N. benthamiana* leaf cells expressing NIa-Pro-GFP or GFP at 48 hpai. DAPI staining indicates the nucleus of tobacco cells, revealing that the green fluorescence of NIa-Pro-GFP is localized not only in the cytoplasm but also in the nucleus. Images (left to right) showed GFP fluorescence, DAPI fluorescence, bright field and overlay of the three images. Scale bars, 10 μm.

Our new supplementation has yielded conclusive evidence through western blot analysis, demonstrating that the fusion protein NIa-Pro-GFP retains its integrity when compared to the control GFP (As shown in revised Fig S14).

**S14 Fig. The presence of intact fusion protein NIa-Pro-GFP in *N. benthamiana* leaf cells was assessed by immunoblotting analysis.** At 48 hpai, infiltrated leaves were sampled for immunoblotting assays using an anti-GFP antibody.

Moreover, an elaborate investigation into the localization of CCP1 was undertaken. Analogous to the observation made of *Arabidopsis thaliana* RD19, a perfect colocalization between CCP1 and Aleurain (a marker for lytic vacuole localization used in the literature) was discerned (revised Fig S15A). Pursuing this inquiry further, we examined the colocalization patterns of CCP1 with endoplasmic reticulum markers. The results underscored a conspicuous colocalization (revised Fig S15B), suggesting that CCP1 is indeed situated on the endoplasmic reticulum membrane initially, and may subsequently be directed to the vacuoles for localization.

**S15 Fig. CCP1 colocalizes with the lytic vacuolar marker AALP (A) and the endoplasmic reticulum marker HDEL (B) in the epidermal cells of *N. benthamiana* leaf.** Images (left to right) showed CFP or GFP fluorescence, RFP fluorescence, bright field and overlay of the three images. Scale bars, 50 μm.

We have conducted additional investigations into the colocalization of CCP1 and NIa-Pro in *N. benthamiana* or maize, revealing that partial colocalization within the cytoplasm occurs in the initial stages following agrobacterium infiltration (prior to 48 hours post-infiltration) or maize leaves (revised Fig S16A, C), whilst in the latter phase of expression (from 48 to 72 hours post-infiltration), colocalization within the vacuoles can be observed in a subset of cells (revised figure S16 B). In the revised manuscript, we have added the section. Please find the detailed information on lines 272-288 of revised manuscript and revised Figs S15 and S16.

**S16 Fig. SCMV NIa-Pro co-localizes with full-length of CCP1.** Confocal images of *N. benthamiana* leave cells co-expressing CCP1-mRFP and NIa-Pro-GFP at 48 (A) and 72 hpai (B). C) The overlay of CCP1-mRFP and NIa-Pro-GFP in the cytoplasm of maize cell at 16 h after bombardment. Images (left to right) showed RFP fluorescence, GFP fluorescence, bright field and overlay of the three images. Scale bars, 50 μm.

Based on these findings, we infer that the localization pattern of CCP1 is similar to that of vacuolar proteases that are synthesized as preproteins in the rough endoplasmic reticulum and transiently transported to the vacuole through the endomembrane secretion system [8], ultimately exerting its protease activity in the vacuole. Furthermore, when co-expressed with CCP1, NIa-Pro is found to be co-localized with CCP1 in the lytic vacuole during the later stage, thereby effectively inhibiting CCP1 activity. We have also incorporated this section into our discussion (Lines 491-495, 497-499).

Related reference:

8.	Lam SK, Tse YC, Miao Y, Li HY, Wang J, Lo SW, Jiang L. Molecular characterization of plant prevacuolar and endosomal compartments. J Integr Plant Biol. 2007;49(8). doi: 10.1111/j.1672-9072.2007.00517.x.

**Minor Issues**

1. Please explain Nla-Pro and Vpg in the introduction.

**Response**：Thanks for your suggestion. We provided explanations for Nla-Pro and Vpg in the introduction section, with specific modifications detailed in the revised main text (on lines 100-106).

2. Please explain to which PLCP class CCP1 belong to.

Reply：Thanks for your constructive comments. The CCP1 belongs to the subfamily 7 of PLCPs, which is homologous to the RD19 gene in *A. thaliana*. We have already provided explanations within the text (on lines 228-229).

3. All gene names (e.g. CCP1 in L118) should be printed in italics.

**Response**：Thanks for the suggestion, we have already made modifications in the revised main text.

4. Please state where DCG-04 was obtained from.

**Response**：The DCG04 (CAS No. 314263-42-8) was purchased from the Medkoo brand (imported from the United States). We have already added the information in the section, with specific modifications detailed in the revised main text (on line 733).

5. I don’t understand that the PLCP activity goes up when there is less CCP1 at 7dpi in Fig1, and why you do not see the suppression of PLCP activity during SCMV infection.

**Response**：Thanks for your concern. Given that the protein bands detected by our CCP1 antibody range in size from 40 to 50 kD, the identified CCP1 proteins correspond to their precursor form (preCCP1), whereas the detected activity of PLCPs indicates their activated state (that is, the proteolytic enzymes in their mature peptide form). Consequently, at 7 dpi, CCP1 protein levels decline while PLCP enzyme activity paradoxically increases. Upon monitoring the enzymatic activity of PLCPs in plants from both the SCMV-infected and mock-treated groups at various time points, we observed that at 10 dpi, the enzymatic activity in the SCMV group was suppressed relative to the control group. This suggests that during the process of viral infection, there might be a regulatory mechanism affecting PLCP enzyme activity.

6. L154 should read: ‘CMV-GUS control plants’.

**Response**：Thanks for the suggestion, we have already made modifications in the text (please refer to the revised line 218).

7. Please check if the catalytic mutant of CCP1 accumulates as its precursor on a western blot. That would explain why it cannot interact with Nla-Pro.

**Response**：Thanks for your comment. The CCP1 catalytic site mutants applied in different interaction systems lack the N-terminal signal peptide and enzyme inhibitory domains. Therefore, their inability to interact is not likely due to their accumulation as precursor forms. In addition, our Co-IP assay in Fig 4C also demonstrate the integrity of the expressed catalytic mutant of mCCP1.

8. L226 I am not convinced that the figures demonstrate that CCP1 is in the cytoplasm. Better images and plasmolysis would be required, supported by WB to show that the fusion is intact.

**Response:** Thanks for your constructive comment. An elaborate investigation into the localization of CCP1 was undertaken. Analogous to the observation made of *Arabidopsis thaliana* RD19, a perfect colocalization between CCP1 and Aleurain (a marker for lytic vacuole localization used in the literature) was discerned (revised Fig S15A, also shown below). Pursuing this inquiry further, we examined the colocalization patterns of CCP1 with endoplasmic reticulum (ER) markers. The results underscored a conspicuous colocalization (revised Fig S15B, also shown in the response to the major comment), suggesting that CCP1 is indeed situated on the ER membrane initially, and may subsequently be directed to the vacuoles for localization.

9. Why does FoMV-3Flag-Nia-Pro expressing fewer heterologous proteins? Are you not comparing two different proteins having the same tag?

**Response**：We employed FoMV to express SCMV NIa-Pro with GFP as the control group. Following FoMV inoculation at 7 dpi, we detected the overexpressed protein levels in maize. However, the results indicated that the accumulation level of NIa-Pro was quite lower than that of GFP. It is speculated that variations in the expression levels of exogenous proteins exist in virus-based vectors.

10. Please explain what type of virus CMV and FoMV are. If they are also potex viruses then this might interfere in SCMV infection.

**Response**：Thank you for this suggestion. CMV (cucumber mosaic virus) belongs to the genus *Cucumovirus* in the family *Bromoviridae*, while FoMV (foxtail mosaic virus) belongs to the genus *Potexvirus* in the family *Alphaflexiviridae*. SCMV falls under the genus *Potyvirus* in the family *Potyviridae*, making CMV and FoMV distinct from SCMV in terms of virus classification. We added this information in line 172 and lines 324-325.

11. How was the structure predicted for Fig6B?

**Response**：The structure in Fig 6B was predicted based on the complete amino acid sequence of NIa-Pro, utilizing the online structure prediction software SWISS-MODEL (https://swissmodel.expasy.org/interactive) in combination with the PDB protein structure database (<https://www.rcsb.org/>). This information is shown in lines 725-729.

12. Please add a reference for the C-terminal inhibitor motif (L284).

**Response**：Thanks for your suggestion, we have already added the references in the revised manuscript (please refer to the revised lines 340-347).

13. Please add explain how likely it is that previously studied PLCPs are not co-silenced in CMV-CCP1 plants.

**Response**：To achieve efficient and specific CCP1 silencing, we conducted an analysis of the full-length gene sequence of CCP1. By aligning the sequences of various *PLCPs* genes in maize and combining the prediction of siRNA efficiency and non-off targeting specific sequences from the website (https://vigs.solgenomics.net/), we ultimately selected a fragment of the CCP1 gene located at the 5' end from 27 nt to 224 nt for silencing (refer to Fig S2). It can be observed that the consistency of the sequence of various *PLCPs* in the region where this fragment is located is very low, indicating that the silencing of CCP1 should be specific. The information is shown in lines 170-171.

14. Are the first two bars in Fig2D and 2F based on the same dataset? This should be disclosed in the legend. Or better would be to remove this from Fig2F to avoid confusion.

**Response**：Thanks for your suggestion. Upon verification, it has been confirmed that the first two columns in Fig 2D and 2F are indeed based on the same dataset. To avoid confusion, we have integrated Fig 2D and 2F, presenting them in a combined form as shown in the revised Fig 2E.

15. Fig3BCD The stats should be added to compare CMV-GUS with CMV-CCP1.

**Response**：Thank you for your suggestion. We have incorporated the statistical analysis of CMV-GUS and CMV-CCP1 in Fig 3B,C, and D. Please refer to the revised Fig 3 for specific results.

16. Were constructs for Fig4 produced with or without signal peptide?

**Response**：In Fig 4, the mature peptide form of the CCP1, i.e. mCCP1, was used for various interplay validations, lacking the signal peptide and enzyme activity inhibitory domain.

17. Please add stats to support Fig5B and 5C.

**Response**：Thanks. We have incorporated statistical analysis in Fig 5B and 5C. Please refer to the updated Fig 5 for specific results.

Reference:

1.	Shindo T, Misas-Villamil JC, Horger AC, Song J, van der Hoorn RAL. A role in immunity for Arabidopsis cysteine protease RD21, the ortholog of the tomato immune protease C14. PLoS One. 2012;7(1). doi: ARTN e29317 10.1371/journal.pone.0029317. PubMed PMID: WOS:000301188800012.

2.	Shindo T, Kaschani F, Yang F, Kovacs J, Tian F, Kourelis J, et al. Screen of non-annotated small secreted proteins of *Pseudomonas syringae* reveals a virulence factor that inhibits tomato immune proteases. PLoS Pathog. 2016;12(9). doi: ARTN e1005874 10.1371/journal.ppat.1005874. PubMed PMID: WOS:000385621900036.

3.	Nunn CM, Jeeves M, Cliff MJ, Urquhart GT, George RR, Chao LH, Tscuchia Y, Djordjevic S. Crystal structure of tobacco etch virus protease shows the protein C terminus bound within the active site. J Mol Biol. 2005;350(1). doi: 10.1016/j.jmb.2005.04.013.

4.	Sun P, Austin BP, Tözsér J, Waugh DS. Structural determinants of tobacco vein mottling virus protease substrate specificity. Protein Sci. 2010;19(11). doi: 10.1002/pro.506.

5.	Ekanayaka P, Weerawardhana A, Chathuranga K, Park JH, Lee JS. Foot-and-mouth disease virus 3Cpro cleaves BP180 to induce blister formation. Viruses. 2022;14(9). doi: 10.3390/v14092060.

6.	Wan L, Wang X, Wang T, Yuan X, Liu W, Huang Y, Deng C, Cao S. Comparison of target pocket similarity and progress into research on inhibitors of picornavirus 3C proteases. Chem Biodivers. 2023;20(3). doi: 10.1002/cbdv.202201100.

7.	Chung BY, Miller WA, Atkins JF, Firth AE. An overlapping essential gene in the *Potyviridae*. Proc Natl Acad Sci U S A. 2008;105(15). doi: 10.1073/pnas.0800468105. PubMed PMID: WOS:000255237200045.

8.	Lam SK, Tse YC, Miao Y, Li HY, Wang J, Lo SW, Jiang L. Molecular characterization of plant prevacuolar and endosomal compartments. J Integr Plant Biol. 2007;49(8). doi: 10.1111/j.1672-9072.2007.00517.x.
