# Supplementary material for: NIa-Pro of sugarcane mosaic virus targets Corn Cysteine Protease 1 (CCP1) to undermine salicylic acid-mediated defense in maize
Source: PLoS Pathog. 2024 Mar 14;20(3):e1012086. doi: 10.1371/journal.ppat.1012086 (PMC10965072; doi:10.1371/journal.ppat.1012086)
Supplement: Attachment — Submitted filename: Response to Reviewers-0222.docx [file ppat.1012086.s027.docx]

**Dear Editor and Reviewers,**

We really appreciate your time and efforts in this manuscript. Your insightful comments and constructive suggestions significantly improved our work for publication.

Please find our point-by-point responses to your comments in below, with our responses highlighted in blue. The corresponding changes are also highlighted in blue in the revised manuscript. Thank you again for your considerations.

With best regards,

**Tao Zhou**

**(on behalf of all co-authors)**

**Point-by-point responses**

**Reviewer #1:**

**Major Issues**

No Response

**Minor Issues**

 1. Line 32: delete “only”, while the interaction of NIa-Pro is convincingly supported by three different protein-protein interaction methods, the initial screening for interaction with other viral proteins may not have allowed the detection of other interactions using the Y2H (nuclear-located) approach.

**Response**: Thanks very much for your suggestion. We have made this correction on line 32.

2. Line 173: change “were served” to “served”

**Response**: Many thanks. We made this correction.

3. Line 199: change “a both significant increasement” to “a significant increased for both”

**Response**: Thanks. We corrected this on line 198 in the revised manuscript.

4. Lines 361, 536, 538: change “230K and 234D” to “K230 and D234”. Please check entire text and figure legends to ensure that there are no other occurrences.

**Response**: Thank you for your suggestions. Accordingly, we checked entire text and figure legends, and have made same modifications in the revised main text (on lines 360, 535 and 537) as well as in the figure legend of S20.

5. Line 395: change “system leaves” to “systemic leaves”

**Response**: Thanks for your suggestion. We corrected it on line 394 of revised manuscript.

6. Line 426: change “increasement” to “increase”

**Response**: Thanks. We corrected this on line 425 of revised manuscript.

7. Fig 4E legend: I appreciate that the His Abs show unspecific binding for the GST protein but it would be useful to add a note to that effect in the legend. Perhaps something like “please note that the His Abs did show unspecific binding for the GST and GST-NIa-Pro proteins”

**Response**: Thank you for your valuable advice. We have incorporated your mentioned clarification in the legend of Fig 4E.

**Reviewer #2:**

**Major Issues**

No Response

**Minor Issues**

1. Abstract: arms races

**Response**: Thanks for your correction. We corrected this in the revised manuscript.

2. Fig. 1B, as explained in the responses by authors, CCP1 exists multiple forms. To avoid misunderstanding by readers in the future, authors should indicate which form of CCP1 supposed to be in the gel, although they may be hard to distinguish because of tiny band size differences. Alternatively, authors can write more clearly in lines 151-156. In the figure legends, authors should also add this information. Please check throughout the manuscript.

**Response**: Thanks for your concern and suggestion. The CCP1 presented in Fig. 1B is referred to as preCCP1 (a full-length protease containing signal peptide and enzymatic inhibitory domains). Following your suggestion, we labeled the gel with preCCP1, and have provided a more explicit description in lines 153-156 of the revised manuscript (also shown below). Additionally, we have included these detailed explanations in the legend of Fig 1.

Immunoblotting with a specific anti-CCP1 antibody indicated a downregulation of CCP1 precursor (preCCP1) during viral infection at 5 and 7 dpi, while an upregulation was observed at 10 and 12 dpi. This pattern suggests potential post-translational regulation of CCP1 in SCMV-infected maize plants.

3. Regarding Fig.6A, strictly, potyvirus has two ORFs, a large polyprotein and PIPO. The large polyprotein is further processed into 10 mature proteins by own proteases, and PIPO is expressed as fusion with the N-terminal of P3 as P3N-PIPO (Vijayapalani et al. 2012, PLoS Pathogens). If authors want to show the 11 proteins encoded by SCMV, then P3N-PIPO is more appropriate, but not PIPO.

**Response**: Thank you very much for your helpful suggestion. We do want to present the 11 proteins encoded by SCMV in Fig 6A, so following your advice, we have changed PIPO to P3N-PIPO. Please refer to the modified Fig 6 for details.

4. S4 Fig, please indicate which CMV gene in RNA 1/2/3 has been used for viral accumulation quantification by RT-qPCR.

**Response**: Thanks for your this suggestion. We added the information that the *coat protein* (*cp*) gene of CMV RNA3 was used for RT-qPCR analysis in the figure legend of S4.

5. S7 Fig, please indicate each band clearly. We can clearly see GUS-3Myc control show band in the FLAG-IP anti-myc gel, suggesting relatively weak interaction signal or this is a unspecific band?

**Response**: Thank you very much for your constructive comments. Following your suggestion, we have marked the corresponding 3Flag-NIa-Pro, GUS-3Myc and preCCP1-3Myc with asterisks in Fig S7 (also shown here). In the GUS-3Myc+NIa-Pro-3Flag sample, the GUS-3Myc control did indeed show a faint band in the FLAG-IP anti-myc gel, which might indicate a relatively weak interaction signal between them.

**S7 Fig.** **Co-immunoprecipitation (Co-IP) assay was conducted to verify the interaction between preCCP1 and NIa-Pro in *N. benthamiana* leaves.** 3Flag-NIa-Pro was co-expressed with preCCP1-3Myc or GUS-3Myc in *N. benthamiana* leaves through agro-infiltration. Leaf tissues were harvested at 48 hours post agroinfiltration (hpai) for Co-IP analysis. Samples from plants co-expressing 3Flag-NIa-Pro and GUS-3Myc were used as a negative control. Co-IP assay was performed using an anti-Flag affinity agarose gel. Protein samples (Input) and the immunoprecipitated protein samples (Flag-IP) were analyzed through immunoblotting assays using an anti-Flag or an anti-c-Myc antibody. *, the protein bands corresponding to the expected 3Flag-NIa-Pro, GUS-3Myc or preCCP1-3Myc.

6. S12 Fig, please indicate N- and C- termini, and more importantly, the mutation residues.

**Response**: Thank you for your suggestion. We have revised the S12 diagram to include the three-dimensional conformation of the mutant proteins with mutated mCCP1 and catalytic triad binding sites. We have also labeled the N-terminus, C-terminus, and their respective catalytic triad binding sites (Please refer to the revised Fig S12 for details, also shown here).

**S12 Fig.** **Three-dimensional model of mCCP1 (A) and mutant mCCP1_C161A, H303A, N330A_ (B), in cartoon style.** The N and C termini of the protein are indicated by black arrows, and the catalytic triad key residues are labeled as yellow (C161), red (H303), and green (N330).

7. S18 Fig is not in order. It appears firstly in the discussion, and should be placed in order. The results shown in S18 Fig is interesting, raising another pathway that CCP1 can suppress SCMV infection. Of course, this does not affect the main conclusion in this MS.

**Response**: Thank you for your suggestion. We have rearranged the order of the S18 figure and updated it as Fig S21. Moreover, we have made the necessary modifications to the figure codes in the main text (on lines 355, 361, 398 and 522).

Your comments remind us for future research on the potential impact of full-length CCP1 on SCMV NIa-Pro protein stability, which may inhibit SCMV infection.

**Reviewer #3:**

**Major Issues**

1. The authors claim that CCP1 was not expressed with a signal peptide for the experiments in Fig4, but Fig4CDE are agroinfiltration experiments, which means that without a SP, CCP1 should accumulate in the cytoplasm and although this might explain why it can interact with cytoplasmic NL1-Pro, this is not the subcellular location of CCP1, given that it has its own SP and given the shown colocalisation with ER and vacuolar markers.

**Response**: Thank you very much for your concern. In Fig 4, we showed that SCMV NIa-Pro interacts with the mature protease domain of CCP1 (mCCP1). Moreover, to validate the interaction between full length CCP1 (preCCP1, with signal peptide) and SCMV NIa-Pro, we performed interaction assays *in vitro* and *in vivo*. The results showed that, although their interaction did not occur in Y2H (Fig S6), preCCP1 could interact with NIa-Pro in both LCI and Co-IP assays employing agroinfiltration (Figs S7 and S8). Since we failed in expressing preCCP1 in *E. coli*, we could not test their direct interaction *in vitro* via GST-pull down. Thus, given that mCCP1 interacted with NIa-Pro in Y2H, LCI, Co-IP and GST-pull down assays, we showed these interaction data as the main Fig 4.

2. The reported data are consistent with a similar study on Nla of WYMV inhibiting RD21 in wheat, which has just been published in NatComm 14:7773. It would be appropriate to discuss the findings in the context of this article.

**Response**: Thank you for your suggestions. We have also reviewed recent research published in NatComm 14:7773, which found an interaction between the NIa protein of WYMV and the RD21 protease of wheat. WYMV and the virus SCMV in this study belong to different genera within the same family, *Potyviridae*. CCP1 identified in this study belongs to the Sf 7 subgroup of PLCPs, while wheat's RD21 belongs to the Sf 1 subgroup. Additionally, the reported data in NatComm 14:7773 revealed that wheat's RD21 can release peptide to activate resistance against WYMV, while WYMV's NIa can facilitate virus infection by interacting with RD21 in susceptible wheat. In our study, we found that CCP1-mediated antiviral activity is associated with the SA pathway, but it is unknown if it directly activates upstream elicitor peptides in the SA pathway. We identified the interaction between SCMV NIa-Pro and CCP1, leading to the suppression of CCP1 activity, and we identified the key residues crucial for the function of NIa-Pro. Following your suggestions, we have added this information to our discussion (lines 503-507, also shown below).

A most recent study uncovered the crucial role of PLCP TaRD21A in wheat against wheat yellow mosaic virus (WYMV, genus *Bymovirus* in the family *Potyviridae*) through the release of a small peptide, while the NIa protein encoded by WYMV suppresses the activity of TaRD21A to facilitate infection in susceptible wheat [86].

**Related reference**

86.	Liu P, Shi C, Liu S, Lei J, Lu Q, Hu H, et al. A papain-like cysteine protease-released small signal peptide confers wheat resistance to wheat yellow mosaic virus. Nature Communications. 2023;14(1). doi: 10.1038/s41467-023-43643-y.

**Minor Issues:** No Response
